# An Integrated Dual Framework for the Design and Reporting of MI and SSVEP Brain–Computer Interface Studies

**DOI:** 10.3390/s26216764

**Published:** 2026-10-26

**Authors:** Nayibe Chio, Eduardo Quiles-Cucarella

**Affiliations:** 1 Institute of Automation and Industrial Informatics—Ai2, Universitat Politècnica de València, 46022 Valencia, Spain; nachch@posgrado.upv.es; 2 Faculty of Engineering, Mechatronics Engineering, Universidad Autónoma de Bucaramanga, Bucaramanga 680003, Colombia

**Keywords:** brain–computer interface (BCI), steady-state visually evoked potential (SSVEP), motor imagery, framework, taxonomy

## Abstract

**Highlights:**

**What are the main findings?**

- An integrated dual framework is proposed to support the systematic design and structured reporting of brain–computer interface (BCI) studies that use MI and SSVEP paradigms for rehabilitation and robotics.
- The framework integrates methodological, technical, and reporting dimensions, promoting consistency, reproducibility, transparency, and comparability in BCI research using MI and SSVEP paradigms for rehabilitation and robotics.

**What are the implications of the main findings?**

- Adoption of the proposed framework can facilitate the development of higher-quality BCI studies and facilitate the interpretation, replication, and documentation assessment of research outcomes.
- The framework provides a common reference model that may contribute to future harmonization efforts and support collaboration among researchers, clinicians, engineers, and other BCI stakeholders.

**Abstract:**

This article presents a framework for brain–computer interfaces (BCIs) based on the motor imagery (MI) and steady-state visually evoked potential (SSVEP) paradigms, with a focus on rehabilitation and robotics applications. The development of this framework was based on a review of scientific literature looking for the best practices, common phases, and key components used in research projects and applications in rehabilitation and robotics. The main result of this work is an integrated and dual framework of 20 components organized into two primary sections: the experimental section and BCI system section. The experimental section formalizes the non-technical phases that are critical for a study’s validity. The BCI system section describes technical architecture. Furthermore, derived from the framework, a taxonomy is proposed to structure language, and a checklist is provided as a practical tool to ensure all the elements are present. The proposed integrated dual framework offers a structure that overcomes the limitations of previous models by merging experimental design with the BCI system’s functional components. Its main contribution is the formalization of all phases of a study, which promotes methodological consistency and facilitates the inclusion of ethical considerations and user characterization. This structure, along with the taxonomy and checklist, provides researchers with a practical guide for designing and reporting BCI studies; documentation assessment in three BCI studies demonstrates the applicability and value of the framework as a practical tool. This contribution aims to promote methodological consistency and comparability among MI and SSVEP-based brain–computer interface (BCI) studies in rehabilitation and robotics.

## 1. Introduction

A framework serves as a guide for researchers to organize and understand the different elements within a study, contributing to its integrity and rigor [1]. Different types of research frameworks exist, like qualitative or quantitative types. The theoretical framework includes the theories that support the research; the conceptual framework includes the concepts involved in the research; the experimental framework includes the procedures for collecting, analyzing, and interpreting data; the methodological framework includes guidelines on the research methods to be used in the study (qualitative, quantitative, or mixed); the analytical or statistical framework includes the techniques and tools for data analysis; and the evaluation framework includes the metrics for assessing the results.

A framework is a structure that provides an organized and reusable foundation for the design, development, implementation, and evaluation of BCI systems. The purpose of the framework is to establish a common vocabulary and a model that can be used by the research community [2]. Several types of frameworks have been identified in the literature:

- A general framework includes components [2,3,4,5] such as the user, signal acquisition, preprocessing to eliminate or minimize noisy signals using filters and techniques to remove artifacts such as eye movements and muscle activity, including band-pass filters, notch filters, etc. [6,7]; feature extraction involves techniques in the time, frequency, spatial, or combined domains, such as Common Spatial Pattern, time–frequency analysis, or autoregressive models [4,6,7,8,9]; feature translation involves converting the extracted features into commands or outputs for the application [6,7,9]; the control interface translates the logical control signals (device-independent) generated by the feature translation into appropriate control signals for a particular type of device, including visual or auditory elements that provide feedback to the user about the system’s state or available options [2,6]; the device controller transforms semantic control signals into physical control signals that act directly on the external device [2,6]; feedback allows the user to observe the outcome of their control attempts and adjust their brain activity accordingly, which can be visual, auditory, or tactile; the user component includes their skills and mental and physical state, and the framework must consider interaction and adaptation to the individual user [2,6,7]; the operating environment is considered in its physical and social context [2,6].
- Some studies establish a common language and methodologies to facilitate the objective comparison of different technologies, which includes the development of benchmarks and standard datasets [2,10]. This also provides methodologies, algorithms and a taxonomy to describe BCI systems related to the target population, tasks, operating environment, BCI control, control interface, and device [2,6], and standardization and comparison components include the comparison of different approaches and technologies through proposed taxonomies, benchmarks, and standardized evaluation methods [2].
- Specific frameworks include a framework for visualization and virtual reality feedback in offline and online analysis [7], a framework for classification using Common Spatial Pattern (CSP) algorithms based on divergence maximization [4] or with autoregressive models [9], a framework for BCI-based games [5], a framework for BCIs in the presence of arbitrary noise sources [11], and a framework for the SSVEP paradigm [10]. However, in addition to these conventional approaches, the transition to integrated BCI systems and commercial applications requires addressing algorithmic problems at different stages of signal processing. To overcome the limitations of traditional methods, particularly in multiclass and noisy scenarios, recent advances have introduced architecture, such as the development of the RW-FBCSP (Riemannian-Weighted Filter Bank Common Spatial Pattern) framework, designed for BCI for MI focused on disability. This approach integrates spatial filtering using CSP, frequency decomposition using filter banks, and covariance matrix calculation using Riemannian geometry, improving feature separation in EEG signals [12].

Brain–computer interfaces (BCIs) capture the signal from the brain’s electrical activity and transform it into a control signal that reflects the user’s intentions, enabling communication between the brain and external devices [13]. BCIs can be invasive or non-invasive. Non-invasive BCIs work with electroencephalography (EEG), measuring the brain’s electrical activity through electrodes placed on the scalp, which makes them safe, portable, and cost-effective [14]. Electroencephalographic (EEG) signals generate specific patterns when using the motor imagery (MI) and steady-state visually evoked potential (SSVEP) paradigms. Motor imagery activates sensorimotor cortical areas during the mental simulation of a movement, while SSVEPs produce a response to repetitive visual stimuli at specific frequencies [15].

Applications of these two paradigms can improve the quality of life for individuals with motor disabilities. In rehabilitation, they help restore motor functions through feedback based on the person’s brain activity [16]. In robotics, they provide a means to control assistive robots, allowing the individual to interact with their environment [17]. In the specific field of robotic applications, recent literature has focused on the development and evaluation of systems dedicated to robot control using BCIs. Non-invasive BCI systems based on EEG signals for controlling robotic arms have been analyzed, comparing the performance and various methods used in the acquisition, preprocessing, feature extraction, classification, and command stages [18]. The feasibility of these systems has been demonstrated by optimizing configuration parameters in three types of MI-based interfaces to control a six-degree-of-freedom industrial robotic arm [19].

Different technologies and methodologies have led to a lack of structured reporting in the design and documentation of BCI studies. Existing frameworks in the literature often focus on the technical components of the BCI system, such as signal acquisition, preprocessing, and classification, while omitting non-technical elements such as experimental design, ethical considerations, and user characterization.

The methodological design used for the development of the framework consists of the following stages:

- Literature Review: Collection of articles and information on frameworks related to the MI and SSVEP paradigms in the areas of rehabilitation and robotics.
- Identification of Best Practices: A sample of 11 general frameworks, 11 SSVEP paradigms and 40 MI paradigms was chosen. From the selected documents, the different phases used in projects implementing the MI and SSVEP paradigms in rehabilitation and robotics were identified.
- Model Development: Based on the identification of best practices, a framework is proposed that includes all the elements used in the development of experiments with the MI and SSVEP paradigms for applications in rehabilitation and robotics.
- Documentation assessment: Documentation assessment was carried out with three studies and a comparative analysis with other studies.

This article aims to propose a framework that includes all the elements that should be considered in experiments and to analyze it in the context of existing frameworks in the scientific literature for the MI and SSVEP paradigms in rehabilitation and robotics applications. This structure, along with a taxonomy and checklist, offers researchers a practical guide for designing and reporting BCI studies, overcoming the limitations of previous models.

The main contribution of this work is based on two areas. The first is integration and methodological consistency, proposing a unified structure for the experimental section and the BCI system, enabling more detailed and replicable experiment design and documentation. The second contribution is a framework that identifies the building blocks in each section, generating a taxonomy and structured checklist aimed at standardizing documentation.

This paper is organized as follows. Section 2 introduces the proposed dual framework for the design and reporting of brain–computer interface (BCI) studies, describing its conceptual foundations, structure, and key components. The methodological approach used to develop and refine the framework is also presented. Section 3 details the application of the framework through representative use cases and examples, illustrating how it supports the planning, execution, documentation, and evaluation of BCI research. Section 4 discusses the implications of the proposed framework, highlighting its potential benefits in guiding researchers in maintaining methodological consistency, reproducibility, transparency, and comparability across studies. Finally, Section 5 summarizes the main conclusions of this work and outlines future directions for the adoption and further development of structured practices in BCI research.

## 2. Materials and Methods

### 2.1. Identification of Existing Frameworks

A new framework is proposed to address all the technical and non-technical elements that should be considered in the development of BCI applications. The proposal was based on a review of 62 articles from the years 2000 to 2026 (July) related to BCI and their frameworks. 

An exploratory review was conducted based on the adaptation of Kitchenham’s methodological guide for its implementation, and the report was structured according to the standards of the Prisma statement; the flow diagram is presented in Figure 1. The PRISMA checklist used to guide and report the exploratory review is provided in the Supplementary Materials (Table S1).

The search for articles was conducted in the SCOPUS database. The keywords used were “Brain-Computer Interface,” “Framework,” “Motor Imagery,” “Steady State Visually Evoked Potentials (SSVEP),” “Rehabilitation,” and “Robotics.” The last search was performed on July 21, 2026, with the search string “framework AND (“Brain computer interface”) AND ((“motor imag*”) OR SSVEP) AND (robot* OR rehab*)”. Based on the search results, inclusion and selection criteria were applied to select articles that would serve as the basis for developing the framework. The review was limited to articles published between 2000 and 2026. The inclusion criteria were articles that presented a framework and were classified as conceptual, applied, research, or algorithmic; studies that used the motor imagery paradigm or SSVEP; and research applied to rehabilitation or robotics. Exclusion criteria were articles unrelated to the SSVEP or MI paradigm, studies that did not propose a framework, and research outside the fields of rehabilitation and robotics. Book chapters, reviews, articles without an author, or articles that could not be accessed were removed. Furthermore, some articles with fewer than five citations or no citations were removed; the citation threshold was applied only to older articles, while recent publications (2026) were assessed independently of the number of citations. The search was conducted in three parts: one on general frameworks and BCI, and two additional searches that included the motor imagery paradigm (SSVEP) with applications in rehabilitation and robotics. After filtering, 62 articles were selected, 11 on general frameworks, 11 on SSVEP, and 40 on MI, to ensure balanced representation. The 62 articles were analyzed based on different phases and components, such as paradigm, approach, application, experimental protocol, testing, regulations, and technical components. The aim was to identify best practices for developing the proposed framework.

Table 1 analyzes these studies, finding the paradigm, approach, application, experimental protocol, testing and regulations, and technical components. The paradigm was divided into three classes: P300, SSVEP, and MI; although BCI research encompasses different paradigms, such as the P300 evoked potential (as shown in Table 1), the scope of the proposed framework is explicitly restricted to MI and SSVEP. This decision stems from the specific physiological and technical requirements of these two paradigms in the context of continuous robotic control and active motor rehabilitation. The approach was divided into four classes: methodology/concepts, application, research, and algorithm. The application was divided into five classes: assistance, rehabilitation, robotics, general, and algorithm development or improvement. The experimental protocol relates to information about the overall process of the experiment, training, and validation. The testing and regulation section covered psychological and physical testing and ethical considerations. The technical component section covered the preprocessing (P), feature extraction (FE), classification (C), and feedback (F) stages.

### 2.2. Proposal for an Integrated Dual BCI Framework

The current review of frameworks focuses on functional components such as data acquisition, preprocessing, feature extraction, classification, device, and feedback, but ignores experimental design and user-centered elements, which are considered non-functional components. The proposed framework includes both functional and non-functional components, aiming for a modular and integrated design that provides general guidance for application development through the application of best practices. Its component-based design allows for continuous integration and flexibility to incorporate new elements, methods, or techniques as the field of brain–computer interfaces evolves.

The proposal involves identifying the blocks with colors to establish a visual reference for brain–computer interface applications, thus creating a functional visual communication strategy. Its aim is to identify the nature of each component, allowing for an integrated interpretation of the diagram, distinguishing between tangible components and processes such as filters or algorithms, and identifying blocks with similar tasks. The following categories are defined: planning, human, physical, processing, learning, and control. Colors are elements of visual language that can convey ideas, messages, and meanings [73]. The colors in Table 2 were selected following conventional associations in engineering and cognitive science to represent the nature of each block. Connecting lines will be black, following engineering conventions for programming. The main flow will be represented with a solid line, while the secondary flow will be represented with a dashed black line [74,75,76].

The proposed framework is based on the functional components found in Table 1 and incorporates additional components related to the experiment and the BCI system (Figure 2). The design logic and selection of exactly 20 components for this framework are derived from fundamental principles, integrating the basic requirements of human subject research with the standard BCI signal processing flow. The goal is to provide an integrated dual BCI Framework, taxonomy and checklist that ensure the inclusion of all elements involved in BCI application development. Figure 2 illustrates the macro-architecture of this complete dual framework, displaying both the experimental and technical sections side-by-side.

#### 2.2.1. Experimental Section

The specific components of the experimental section are described below. As isolated from the general framework (left side of Figure 2), this specific section consists of 8 non-technical components (Figure 3): recruitment, preparation and information, initial tests, training session, validation session, additional tests, experiment completion, and timeline. Figures for each component are found in Appendix A. 

##### Recruitment and Preliminary Activities

This component (Appendix A—Figure A1) relates to how participants are recruited (e.g., flyers, brochure, email, website, in-person talks) and the preliminary activities, such as obtaining ethical committee approval, reviewing existing laws such as the Helsinki Declaration of 1964, formulating the informed consent document, and printing necessary documents before the experiment, such as instructions and experiment information (name of the experiment, contact information, location, description of the experiment, background information, risks and withdrawal).

##### Preparation and Information

This component (Appendix A—Figure A2) covers activities performed on the day of the experiment, including equipment activation and connection checks, participant setup, electrode verification, and signal recording. Participants must be informed about the experiment and the emergency protocol in case they wish to withdraw or stop, or an emergency requiring first aid occurs. Finally, the participant must sign the consent form.

##### Initial Tests

Initial tests (Appendix A—Figure A3) are related to the physical or mental condition of the participant and their experience with BCI systems. These may include an initial questionnaire covering physical exercise, video gaming, musical training, a demographic survey, psychological tests, or relaxation exercise, and a question about the user experience with the BCI.

##### Training and Validation Session

This session (Appendix A—Figure A4) is performed on the BCI system and includes data recording, processing, offline training, model validation, model adjustment, model inclusion, online experiments, and analysis. 

##### Additional Tests

Additional complementary tests, such as Nesplora Classroom for virtual reality or CPT II (Conners Continuous Performance Test II), can be conducted to formally assess specific user cognitive states, including general attention capabilities, concentration, and alertness, and it also offers a score for the participant’s ability to inhibit a response [77]. 

##### Experiment Completion

This stage (Appendix A—Figure A5) involves removing the user interface, applying a perception survey about the experiment, and verifying that all data has been saved correctly. 

##### Experiment Timeline

The experiment timeline (Appendix A—Figure A6) covers the entire procedure from the training and validation session, which typically requires a total of 70 to 90 min. A standard protocol allocates approximately 10 min for preparation and informed consent, 10 min for initial testing, 30 to 50 min for the core BCI experiment (training and validation), 15 min for complementary tests, and 5 min for the final completion phase.

#### 2.2.2. BCI System Section

The BCI system is based on the model proposed by Mason in 2003, in which a user controls a device through functional components [2]. The proposed BCI system (Figure 4) is implemented during the training and validation session. This proposal includes 12 functional components: paradigm selection, stimulus, environment, user, interface, data acquisition, preprocessing, feature extraction, classification, command, device, and feedback. Figures for each component are found in Appendix B.

##### Paradigm Selection

Paradigm selection (Appendix B—Figure A7) relates to the tasks the BCI user must perform to represent their intentions, which are captured from their brain signals in specific brain regions [78]. BCI systems can employ invasive or non-invasive modalities [79]. This framework focuses on non-invasive EEG as it is easy to use, portable, and affordable [80]. Specifically, it focuses on two paradigms: Steady-state visual evoked potentials (SSVEP) are derived from the brain’s response to visual stimulation, with maximum amplitude in the occipital region [81,82], with advantages such as simple setup, high speed, many available commands, high reliability, and minimal user training [23]. Motor imagery (MI) is driven by the mental simulation of body movements [83]. 

The sampling rate should be at least double the highest frequency of interest, according to the Nyquist theorem [29,84]; the framework defines the brain regions and the relevant EEG frequency bands. The system specifically focused on Alpha (8 to 12 Hz) and Beta (12 to 30 Hz). 

##### Stimulus Type

The stimulus type (Appendix B—Figure A8) depends on the chosen paradigm. It can be visual for SSVEP or self-regulated for MI, which dictates the operational strategy (selective attention or cognitive effort) [79]. The stimulus generation method (software or hardware) and the display device are also important. For SSVEP protocols, researchers must configure specific visual characteristics (e.g., color, size, shape), display hardware, and frequency bands, while actively mitigating risks such as eye strain or epileptic seizures may occur [82]. Regardless of the paradigm, the framework requires establishing precise temporal parameters for experimental tasks, which includes detailing the signal sequences, the duration of the stimuli (e.g., 1 to 10 s), the intervals between stimuli (e.g., 0.1 to 1 s), and the rest periods (e.g., 1 s to 5 min). 

##### Environment

The environment (Appendix B—Figure A9) is the location where the experiment takes place. It includes the user’s position (who should be comfortable and still), the distance between the user and the stimulus (e.g., 60 cm), and the ambient conditions (location, noise, and lighting).

##### User

The user (Appendix B—Figure A10) controls the BCI system and is a variable component due to their physical and mental state. This component includes characterizing the participant group (age, gender, education level, occupation, geographic location, etc.) and identifying human factors like motivation and fatigue through initial tests. 

##### Interface

The interface (Appendix B—Figure A11) is the device used to capture brain signals, including electrodes and an amplifier. Key considerations are the number of electrodes, connection quality, their placement (e.g., 10–20 system), electrode type (wet or dry), and sampling rate. 

##### Data Acquisition

Data acquisition (Appendix B—Figure A12) involves capturing, amplifying, and digitizing the brain’s electrical activity. This includes hardware (the interface and stimulus visualization) and software for data recording, processing, and stimulus generation (e.g., OpenVibe, BCI2000, MATLAB, Python, Lab Streaming Layer (LSL), and BCILab among others). However, for these platforms to be viable in daily use, they must integrate solutions to the problems of the BCI system architecture, such as reducing or eliminating calibration times, improving performance, and mitigating BCI illiteracy [85].

##### Preprocessing

EEG signals can be contaminated by biological or environmental noise [82]. Preprocessing (Appendix B—Figure A13) may include removing bad channels, artifact removal, frequency filtering, baseline correction, and data normalization. The framework involves the rejection of physiological and extra-physiological artifacts [79]; established techniques such as Independent Component Analysis (ICA), Empirical Mode Decomposition (EMD), Common Average Reference (CAR), or Principal Component Analysis (PCA) can be used for removal. Furthermore, it requires the configuration of spatial and frequency filters [82], Laplacian Spatial Filtering [86], notch (typically for 50/60 Hz power line interference) or specific FIR (Finite Impulse Response) for offline processing or IIR (Infinite Impulse Response) for online processing.

Static filters such as FIR and IIR are used in EEG preprocessing, but they fail to account for the intrinsic interindividual variability of neural rhythms. To overcome this limitation, subject-specific adaptive filtering has emerged as an alternative. A practical hardware implementation has demonstrated that using subject-specific adaptive filters during the training phase increases classification accuracy in online scenarios [87]. Furthermore, for embedded BCI systems, advanced dynamic filtering techniques such as the weighted overlapping sum algorithm (WOLA) have been proposed. The WOLA method acts as a bank of adaptive filters that dynamically isolates subject-specific frequencies by analyzing vent-related desynchronization and synchronization (ERD/ERS) phenomena. This approach maximizes pattern recognition accuracy and provides a solution for real-time hardware implementations, such as FPGA platforms [88].

##### Feature Extraction

Feature extraction (Appendix B—Figure A14) transforms the electrical signals into feature values that help discriminate the user’s intent [89]. Techniques include [90,91,92] time-domain methods, like statistics or autoregressive models; frequency-domain methods, like Fast Fourier Transform (FFT), Power Spectral Density (PSD) or Band Power; time–frequency methods, like Wavelet Transform or Hilbert–Huang Transform; spatial methods, like Common Spatial Patterns (CSP); non-linear methods, like entropy; and multivariate statistical methods, like canonical correlation analysis (CCA).

##### Classification

The classification stage (Appendix B—Figure A15) uses machine learning techniques to identify the user’s intent from the extracted features [79]. The framework supports a wide range of techniques [93]. Researchers must report on their chosen approach, including standard supervised classifiers such as support vector machines, Naive Bayes, K-nearest neighbor, linear discriminant analysis (LDA) [94], quadratic discriminant analysis (QDA) [94], unsupervised clustering, or advanced Deep Learning. Finally, the model’s performance must be evaluated using metrics like accuracy, recall, F1-Score, confusion matrix, or Information Transfer Rate (ITR) [95].

##### Command Translation

Command translation (Appendix B—Figure A16) converts the classified user intention into a specific command to control the device.

##### Device

The device (Figure 5) is an application that the user interacts with the BCI. The BCI is a communication and control system based on brain activity that allows a person to communicate their intent and act on an environment [90,96]. 

Applications exist in medicine, including wheelchairs [97], robotic arms [98], neuroprosthesis [99], and rehabilitation assistance. In smart environments, there would be applications in security and authentication or home automation systems [100]. In marketing, there would be applications in neuromarketing and advertising. Neurofeedback is related to cognitive neuroscience, including the prevention and diagnosis of brain-related health problems, rehabilitation, and restoration [29]; education or cognitive training; entertainment, such as video games or virtual reality; and communication and control systems, such as spellers [101].

##### Feedback

Feedback is how the user verifies whether their intention was successful and allows them to make adjustments to improve their control over the device. 

## 3. Results

### 3.1. Illustrative Studies of the Integrated Dual Framework for Brain–Computer Interfaces

The proposed integrated dual framework for brain–computer interfaces has been applied in three illustrative studies: two with motor imagery (MI) and the other with steady-state visual evoked potentials (SSVEP). 

The aim of study 1 is to develop a low-cost robotic guide system controlled by a motor imagery BCI for assisted arm rehabilitation [102]. The research demonstrated a practical application of the motor imagery paradigm in a rehabilitation system. The purpose of this study is to demonstrate that the proposed framework formalizes the BCI system components and the experimental process, illustrating the usefulness of the framework to guide the documentation of rehabilitation research.

The aim of study 2 is the implementation of a BCI based on steady-state visual evoked potential for the control of a robotic arm [103]. The purpose of this study is to demonstrate its feasibility in the control of robotic devices, and the purpose of applying the framework is to demonstrate its application to different BCI paradigms, allowing for the documentation of information on technical and experimental components.

The aim of study 3 is to evaluate the performance and user experience of a BCI-controlled robotic system for assisted arm and forearm rehabilitation [104]. The study’s aim is to highlight the importance of human factors and user experience, in addition to the technical aspects of the BCI rehabilitation system. The study assessed the framework on non-technical aspects, such as ethical considerations and experimental protocols. 

### 3.2. Taxonomy and Checklist of the Integrated Dual Framework for Brain–Computer Interfaces

From the proposal for an integrated dual BCI framework, a taxonomy and a checklist are proposed. The taxonomy allows grouping elements that share similar characteristics, in such a way that a classification and organization system of information related to the components of the experimental section and BCI system is described, providing a common language and a structure for reporting applications with BCIs. 

The taxonomy and checklist have been integrated into Table 3. This unified tool provides researchers with the standard terminology (taxonomy), with practical verification steps (checklist) for each of the 20 components of the framework. The domain of study is the experiment and BCI system sections. The functional category groups framework elements into macro-phases; the component identifies the element being addressed and defines those components. The taxonomy parameters provide a structured vocabulary and specific variables that researchers must establish and report, while the checklist translates these parameters into practical questions to guide the documentation process.

The documentation of taxonomy and checklist in the three studies is presented below (Table 4).

The documentation demonstrates that the proposed taxonomy and checklist are effective tools for designing and reporting documentation in brain–computer interface (BCI) studies. The framework is not simply a list of components, but rather a structured guide that allows for identifying key elements included or omitted in research reports.

## 4. Discussion

Existing BCI frameworks often focus on technical aspects and neglect stages like experimental design and user-centered elements. The proposed integrated dual BCI framework addresses these gaps in its “Experiment” section. While the frameworks in Table 1 concentrate on technical components, our proposed framework includes elements for recruitment, testing, and informed consent, which are critical for the design and application of the experiment. The proposed framework is dual and integrated because it integrates experimental lifecycle management with the technical design of the BCI system for MI and SSVEP paradigms and provides a taxonomy and checklist within each component. This framework is also flexible, allowing for the incorporation of new elements, methods, or techniques as the BCI field evolves, such as new learning algorithms, artifact removal methods, training protocols, or guidelines related to BCI. For rehabilitation and robotics applications using MI and SSVEP, the components of the “Experiment” section allow for assessing user variability and evaluating cognitive states like fatigue or attention to improve training. The aim of the integrated dual BCI framework is to provide a general guide for developing applications with the best practices. Its component-based design allows for the continuous integration of innovations. Future studies could focus on validating this framework in other application domains.

The integrated dual framework was applied in three studies in rehabilitation and robotics using MI and SSVEP. We recommend assessing it with other types of applications and paradigms to assess its generalizability or determine if adaptations are needed for specific contexts.

The integrated dual BCI framework is divided into two sections and 20 components, while most of the reviewed studies focus on the technical aspects of the BCI system. In contrast, this framework formalizes and structures all the preceding and subsequent phases, such as user recruitment, informed consent, initial and supplementary tests, and experiment completion. 

To eliminate circular bias and assess the objectivity of the framework, independent evaluation was conducted on three external MI and SSVEP studies applied to robotics and rehabilitation. These specific studies were selected as the most cited articles in this field for the years 2024, 2025, and 2026. The selected studies were "Transfer learning for subject-independent motor imagery EEG classification using convolutional relational networks” (Article 1) [52], “A hybrid BCI combining SSVEP and EOG and its application for continuous wheelchair control” (Article 2) [105], and “Discriminative Adversarial Network Based on Spatial-Temporal-Graph Fusion for Motor Imagery Recognition” (Article 3) [47].

To ensure objective assessment, the framework’s checklist employs a binary scoring system (Yes/No). A “Yes” is awarded only if the study provides sufficient detail for independent replication of that specific component; otherwise, it is scored as “No”. It is important to clarify that a “No” rating does not indicate methodological flaws or low scientific quality. For example, in studies focused on algorithms that exclusively use pre-existing public datasets, elements related to initial recruitment or informed consent will be rated “No,” as those phases fall outside the scope of the article. Thus, the binary checklist functions as a comprehensive mapping tool that identifies which segments of the brain–computer interface (BCI) experimental lifecycle are documented.

The checklist was applied independently by two raters from our research group: a senior BCI researcher (Rater 1) and a junior researcher with domain expertise in BCI paradigms (Rater 2). Applying the 20-item checklist to the three articles generated a total of N = 60 observations per rater. Rater 1 recorded 39 “Yes” and 21 “No” responses, while Rater 2 recorded 30 “Yes” and 30 “No” responses. Both raters coincided exactly on 51 of the 60 observations, resulting in an observed agreement of *Po* = 51/60 = 0.85 (85%).

The expected agreement by chance (*Pe*) was calculated based on the marginal probabilities of each response. The probability of an expected “Yes” agreement was 0.325, and the probability of an expected “No” agreement was 0.175, a total expected agreement of *Pe* = 0.5. The inter-rater reliability was calculated using Cohen’s Kappa index (Equation (1)), yielding a score of K = 0.7, which indicates inter-rater agreement.

(1) $$k=\frac{\mathrm{Po}-\mathrm{Pe}}{1-\mathrm{Pe}}=\frac{0.85-0.5}{1-0.5}=0.7$$

By achieving this level of consensus between a senior and junior researcher, the evaluation confirms that the checklist’s taxonomy and criteria can be reliably applied by researchers with varying levels of experience. This statistical validation demonstrates that the framework categories can be interpreted and applied with consistent reliability by different researchers. 

The final evaluation presented in Table 5 reflects blinded raw scores from both evaluators, illustrating how the framework maps and identifies methodological reporting gaps in current cutting-edge literature. 

When comparing the proposed integrated dual framework with established BCI standards such as IEEE P2731 [106] and IEEE P2794 [107], a distinction must be made: while existing standards serve as overarching guidelines defining what information should be reported, the proposed framework functions as an operational tool guiding how to systematically design and report that information.

IEEE P2731 establishes a functional BCI model defining architecture and unified terminology. Rather than replacing it, the proposed framework builds upon this foundation by integrating human, cognitive, and experimental management factors into a 20-component structure. Similarly, IEEE P2794 outlines the essential reporting requirements for in vivo neural interface research. To support alignment with these principles, the framework provides practical management tools—such as an itemized checklist, a visual communication strategy, and a specific taxonomy—designed to facilitate transparent and replicable scientific reporting. 

Furthermore, while foundational models like Mason & Birch [2] defined the standard technical pipeline (acquisition, processing, application), and projects like TOBI [108] and BNCI Horizon 2020 [109] focused on hardware interoperability and broad community roadmaps, respectively, they lack a step-by-step reporting checklist for researchers. The specific increment of the proposal is mapped in Table 6.

The proposed integrated dual BCI framework distinguishes itself from existing brain–computer interface protocols and standards by integrating both technical architecture and the full lifecycle of experimental management. While traditional protocols like Graz-BCI [110] focus on synchronous technical tasks, our framework introduces 20 components that incorporate human and non-technical factors, elements typically absent from Graz-BCI.

Compared to international standards such as IEEE P2731 and IEEE P2794 [107], which serve as guidelines for terminology and reporting, our framework acts as a practical tool for the design and execution of experiments. 

This framework builds upon the conceptual foundation proposed by IEEE P2731 and the model of Mason and Birch [2] by actively formalizing non-technical experimental phases. To support alignment with the transparency and replicability principles outlined in IEEE P2794, the framework offers practical management tools, including a taxonomy, visual communication strategies, and an itemized checklist.

Finally, unlike collaborative initiatives such as TOBI [108] and BNCI Horizon 2020 [109], this framework provides an actionable, step-by-step approach that structures user characterization, ethical protocols, and adaptable management tools for MI- and SSVEP- based BCI systems in robotics and rehabilitation.

The purpose of this integrated dual BCI framework, along with its taxonomy and checklist, is to encourage researchers to consider not only the technical aspects but also the human and logistical context, thereby facilitating a pathway toward improved validity, ethics, and reproducibility of BCI studies.

## 5. Conclusions

The proposed BCI framework offers a structured, dual, and integrated approach that covers the experimental design as well as the functional and non-functional components of a BCI application, utilizing MI and SSVEP paradigms in robotics and rehabilitation applications. By providing structured vocabulary and assessment criteria, this framework hypothesizes a pathway toward more reproducible applications, laying the groundwork for future evaluations of BCI research quality. Previous models only addressed the functional components, this article includes non-functional components such as recruitment, user characterization, study planning, user-experience elements, and ethics.

The contributions of this proposal are the integration of the experiment and the BCI system, a detailed guide for experiment setup, its specific application to MI and SSVEP paradigms in rehabilitation and robotics, and its adaptability to new technologies. 

The framework’s block diagrams represent the workflow, taxonomy table structures, components and design choices, and the checklist helps identify the inclusion of key elements in a BCI application. The taxonomy establishes consistent terminology across all MI and SSVEP-based BCI projects, and the checklist may facilitate reproducibility and comparability between studies.

Documentation assessment in three BCI studies and comparison with other studies demonstrated the applicability and value of the framework as a practical tool compared to general frameworks and articles using the motor imagery paradigm and SSVEP. It is important to note that while the proposed framework, taxonomy and checklist are grounded in established clinical and engineering principles, their actual impact on the BCI community remains hypothetical. Current illustrative applications demonstrate their feasibility but do not provide empirical evidence of improved study quality. Future work should include studies that quantitatively assess whether research documented using this framework shows statistically significant improvements in reproducibility and comparability compared to traditional reporting methods.

The main contribution of this article is an integrated dual framework that includes functional and non-functional components, the structuring of a taxonomy and checklist for the design and reporting of MI and SSVEP-based brain–computer interface (BCI) studies in rehabilitation and robotics, the adaptation of best practices for these specific paradigms, and the practical documentation assessment of the framework.

## Figures and Tables

**Figure 1:**
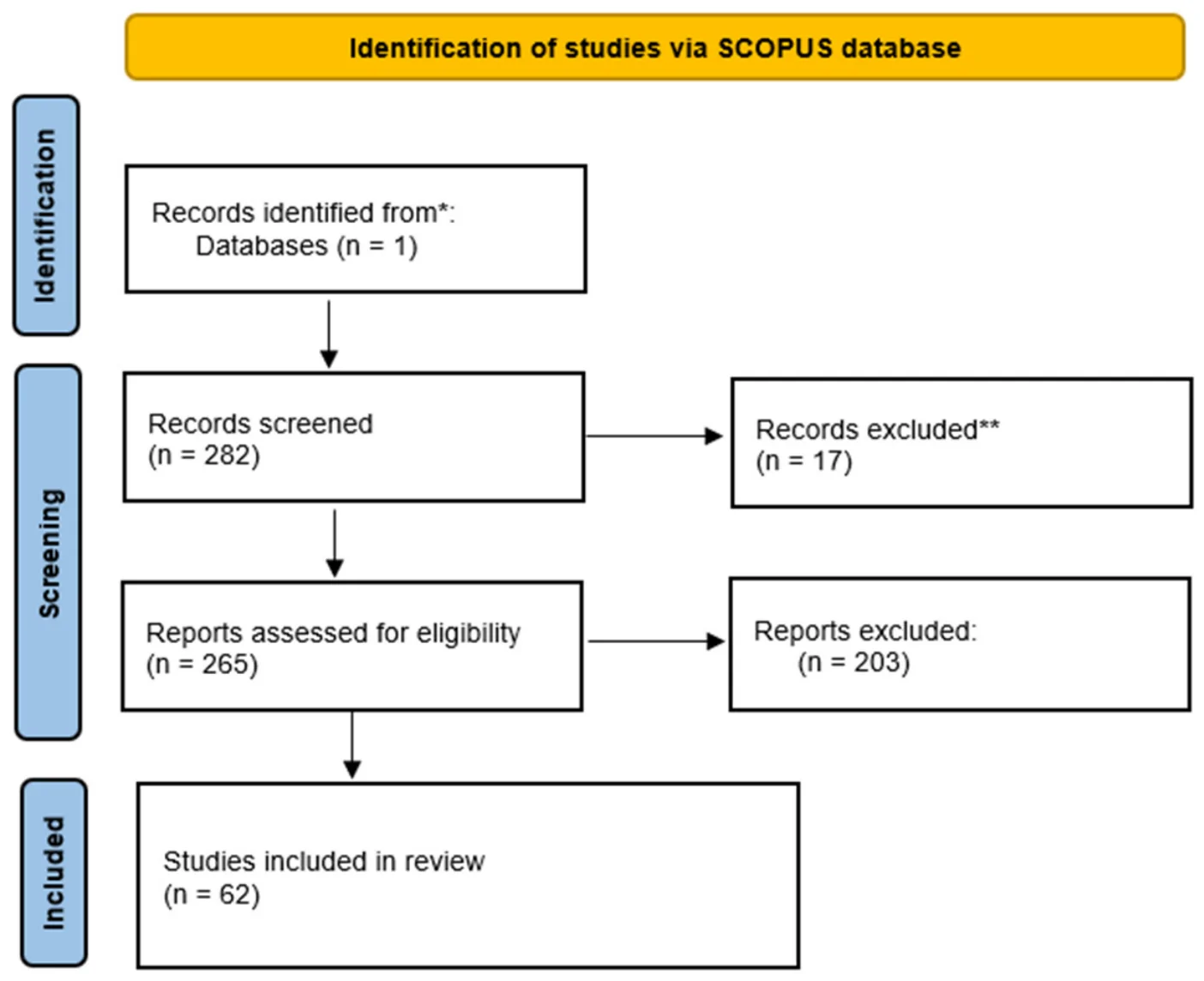
Flowchart of exploration and article analysis.

**Figure 2:**
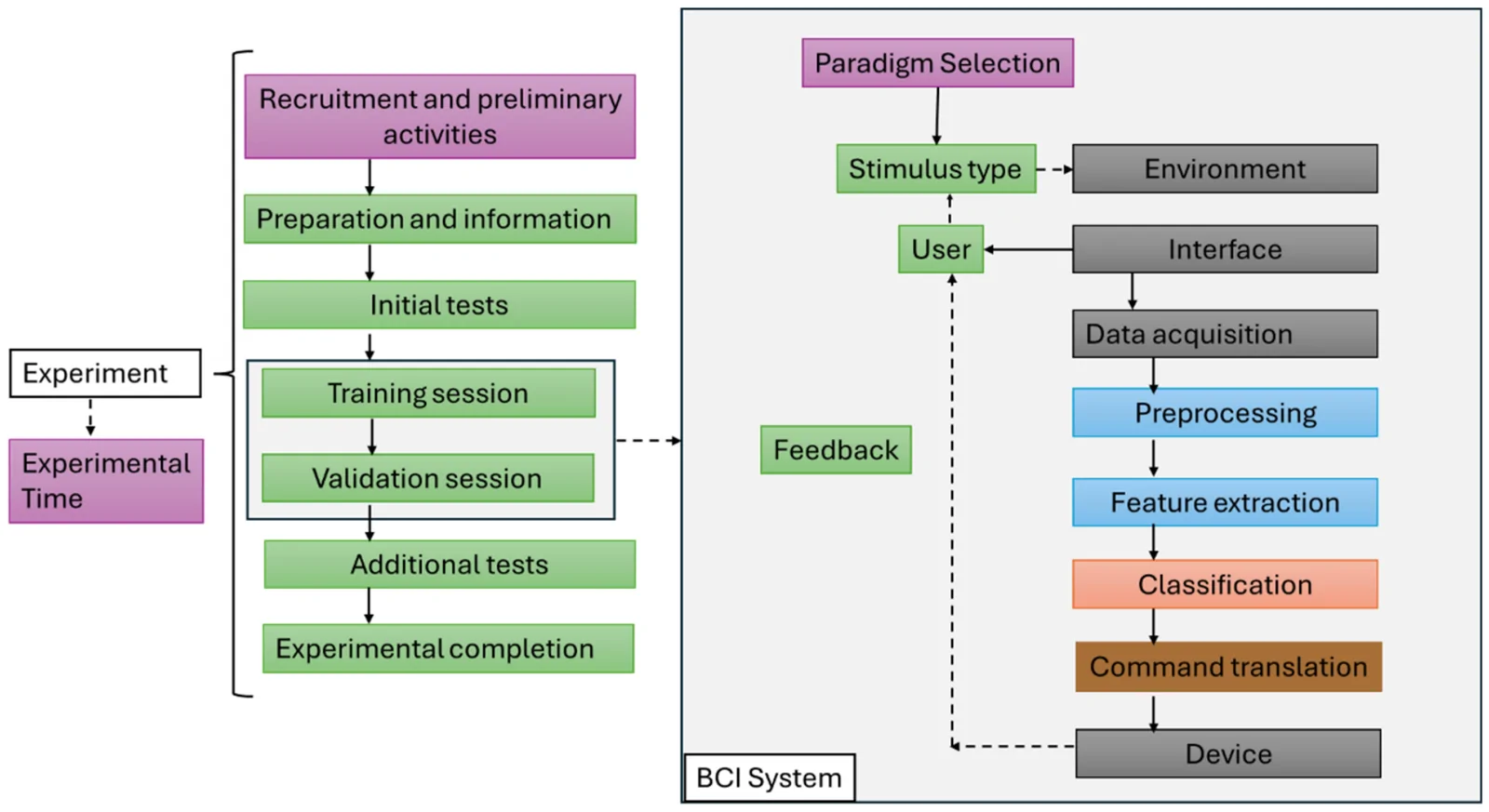
The complete dual framework for BCI applications, illustrating the integration of the experimental section (**left**) and the BCI system section (**right**).

**Figure 3:**
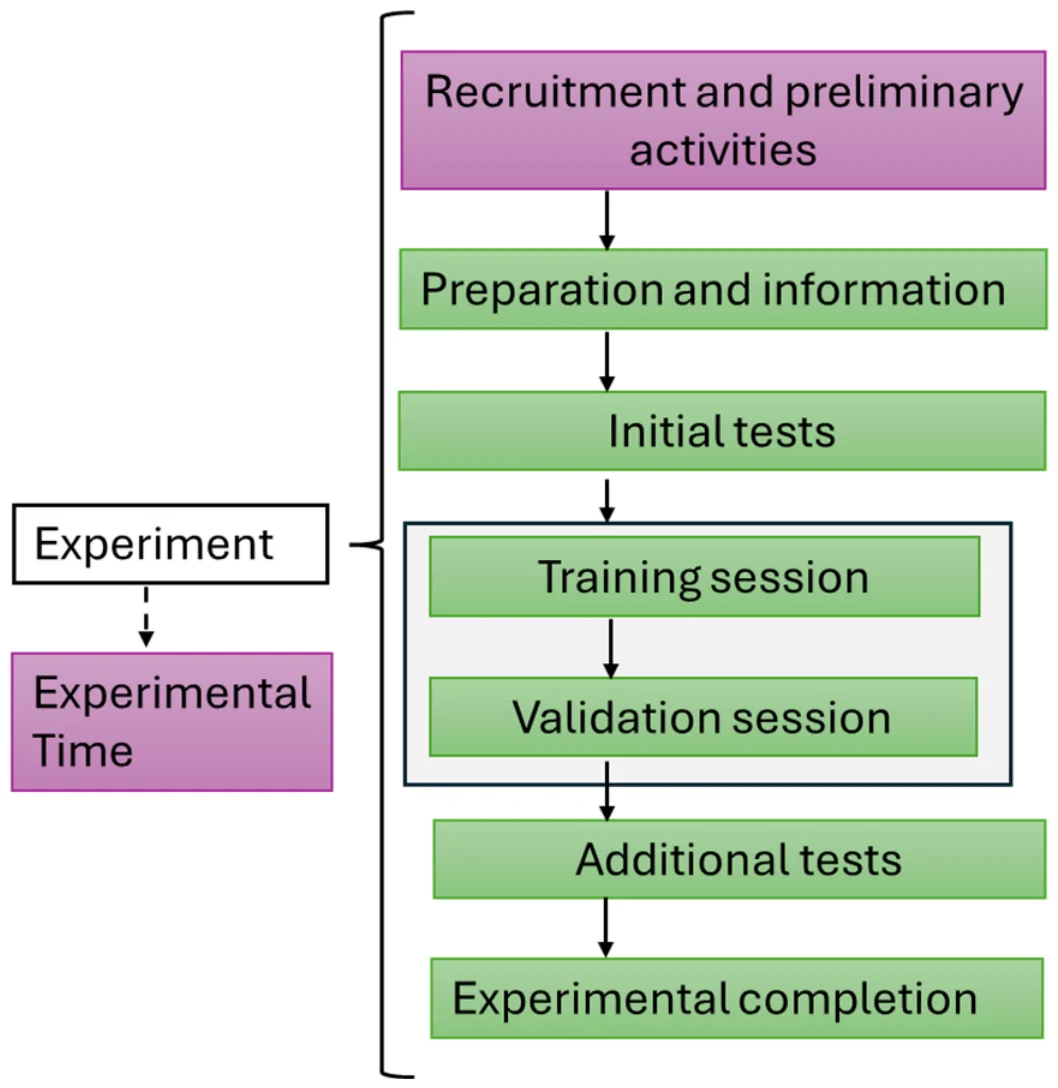
Detailed and isolated view of the experimental section components.

**Figure 4:**
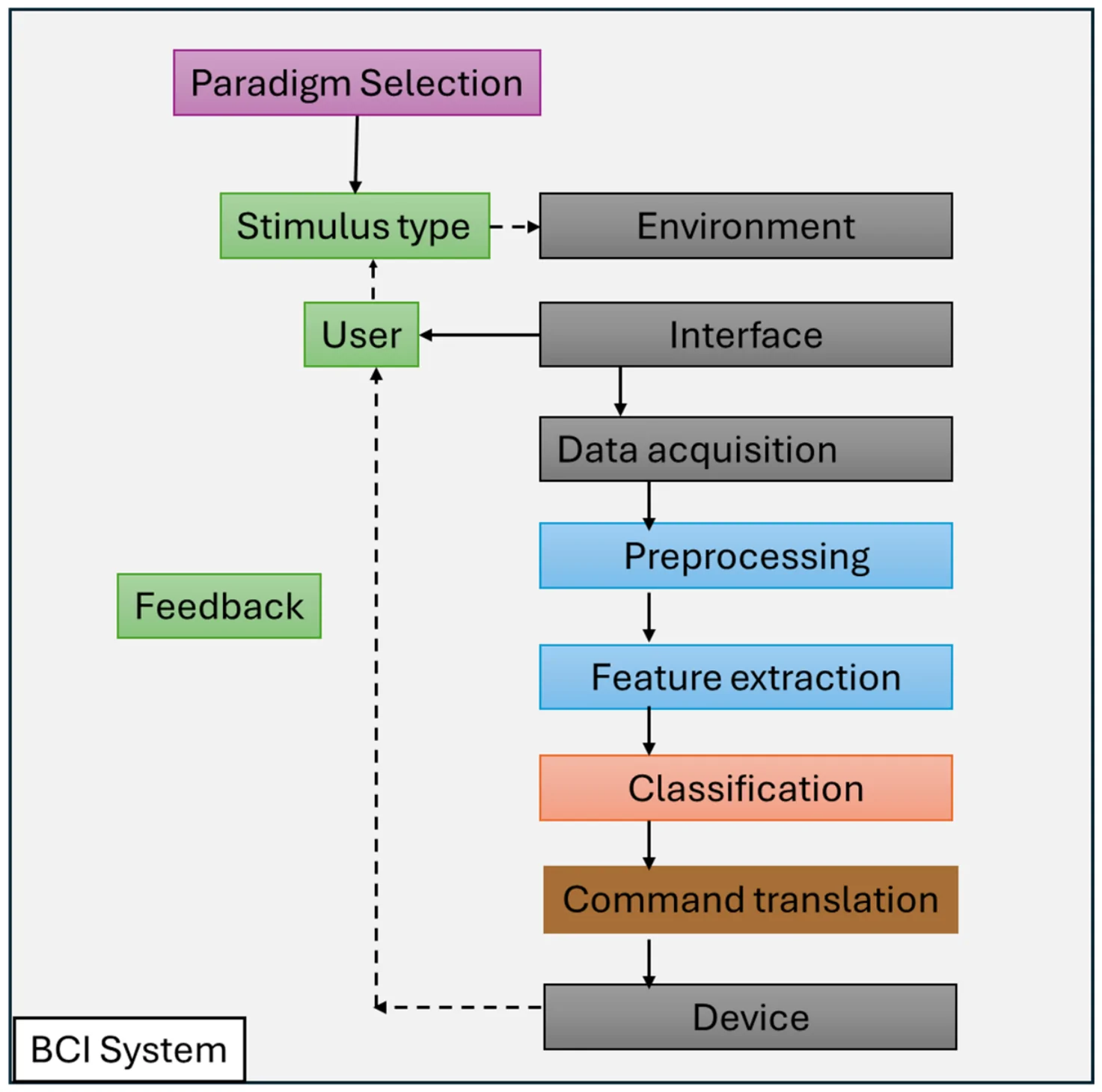
Detailed and isolated block diagram of the BCI system model.

**Figure 5:**
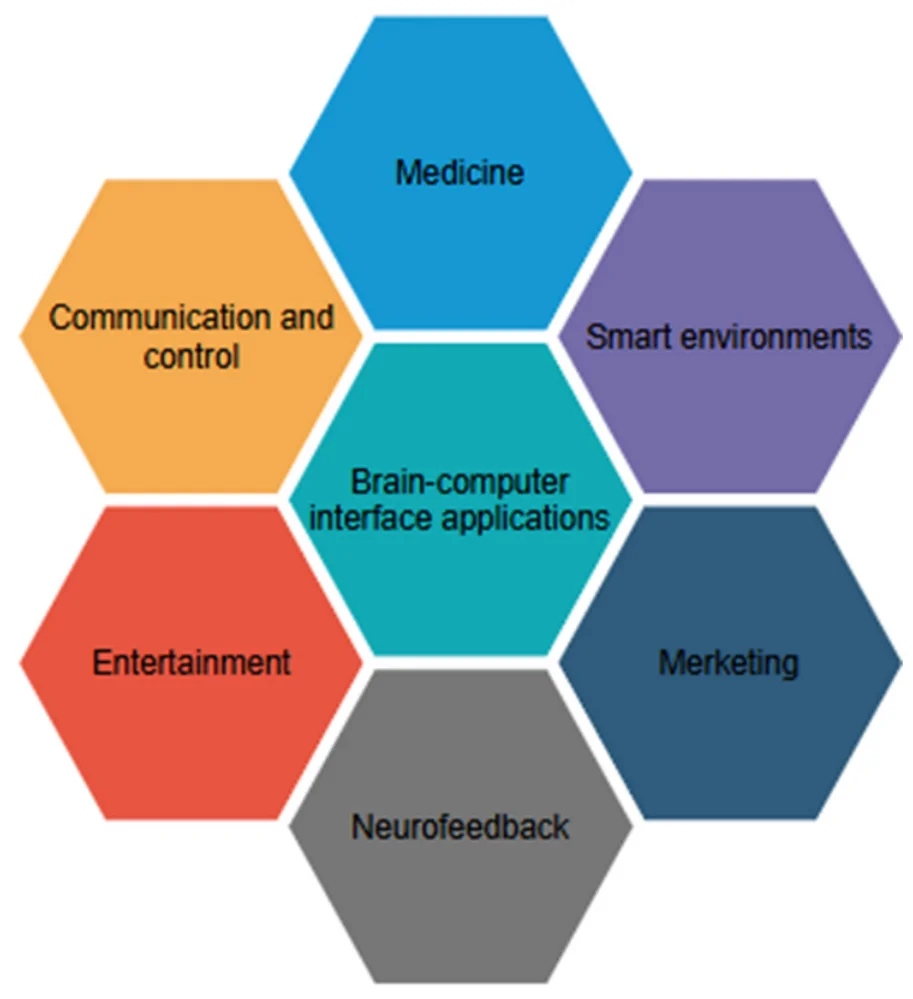
Application areas for BCIs.

**Table 1:** Studies on BCI frameworks.

Study	Paradigm	Approach	Application	Experimental Protocol	Testing and Regulations	Technical Components
[20]	MI, SSVEP, P300	Methodology/ Concepts	Robotics	No information	Human factor, assessment/preprocessing	P, FE, C, F
[21]	MI, SSVEP, P300	Methodology/ Concepts	Assistance	General protocol	Mentions the need to address factors that affect the user	P, FE, C, F
[2]	MI, SSVEP, P300	Methodology/ Concepts	Assistance	No information	No information	P, FE, C, F
[7]	MI	Application	Robotics	Online protocol	No information	P, FE, C, F
[22]	MI	Research	Rehabilitation	Online protocol	Informed consent, clinical examination	P, FE, C, F
[23]	SSVEP	Research	General (Virtual keyboard)	Online protocol	No information	P, FE, C, F
[5]	MI	Application	General (video games)	General protocol	No information	P, FE, C
[9]	MI	Algorithm	Algorithm development or improvement	Dataset protocol	No information	Preprocessing, feature extraction, classification
[6]	MI, SSVEP, P300	Methodology/ Concepts	Robotics	No information	No information	P, FE, C, F
[4]	MI	Algorithm	Algorithm development or improvement	Dataset protocol	No information	P, FE, C
[10]	SSVEP	Algorithm	General (virtual keyboard)	General protocol	No information	F
[24]	MI	Application	Robotics	Online protocol	Ethics approval, skill test	P, FE, C, F
[25]	SSVEP	Application	Rehabilitation	Online protocol	Ethics approval, informed consent, question about BCI experience	P, FE, C, F
[11]	MI	Methodology/Concepts	General	No information	No information	P, FE, C, F
[26]	MI	Research	Rehabilitation	Online protocol	Ethics approval, informed consent, motor testing	P, FE, C, F
[27]	SSVEP	Application	Robotics	Online protocol	Ethics approval, informed consent	P, FE, C, F
[28]	MI	Methodology/ Concepts	Robotics	Protocol Synchronous protocol based on Graz BCI	Ethics approval	P, FE, C
[29]	SSVEP	Application	Robotics	Offline protocol	No information	P, FE, C
[30]	MI	Application	Robotics	Offline and online sessions	Ethics approval, informed consent	P, FE, C, F
[31]	SSVEP	Application	Robotics	Online and offline protocol	Informed consent	P, FE, C, F
[32]	MI	Application	Robotics	Offline protocol	Ethics approval	P, FE, C, F
[33]	SSVEP	Application	Robotics	Online protocol	No information	P, FE, C, F
[34]	MI	Algorithm	Rehabilitation	Dataset protocol	No testing applicable due to dataset use	P, FE, C
[35]	SSVEP	Algorithm	Robotics	Online protocol and dataset protocol	Informed consent	P, FE, C, F
[36]	MI	Algorithm	Rehabilitation	Online protocol	No information	P, FE, C
[37]	SSVEP/Hybrid	Research	Robotics	Offline and online protocol	Ethics approval, informed consent, test to measure mental workload	P, FE, C, F
[38]	MI	Algorithm	Rehabilitation	Online protocol	Ethics approval, informed consent, motor, and cognitive testing	P, FE, C
[39]	SSVEP	Methodology/ Concepts	Algorithm development or improvement	Dataset protocol	No information	P, FE, C
[19]	MI	Algorithm	Robotics	Online and offline protocol	Ethics approval	P, FE, C, F
[40]	SSVEP/Hybrid	Research	Robotics	Offline protocol	Assesses mental workload	P, FE, C, F
[41]	MI	Algorithm	Rehabilitation	Dataset protocol	No testing applicable due to dataset use	P, FE, C
[42]	MI	Algorithm	Rehabilitation	Dataset protocol	No testing applicable due to dataset use	P, FE, C
[43]	MI	Algorithm	General	Dataset protocol	No testing applicable due to dataset use	P, FE, C, F
[44]	MI	Algorithm	Rehabilitation	Online protocol and dataset protocol	Ethics committee and informed consent	P, FE, C
[45]	MI	Algorithm	Rehabilitation	Dataset protocol	No testing applicable due to dataset use	P, FE, C
[46]	MI	Algorithm	General	Dataset protocol	No testing applicable due to dataset use	P, FE, C
[47]	MI	Algorithm	General	Dataset protocol	No testing applicable due to dataset use	P, FE, C
[48]	MI	Methodology/ Concepts	General	Dataset protocol	No testing applicable due to dataset use	P, FE, C
[49]	MI	Algorithm	General	Dataset protocol	No testing applicable due to dataset use	P, FE, C
[50]	MI	Algorithm	Rehabilitation	Offline and online protocol	Clinical examination	P, FE, C
[51]	MI	Algorithm	Algorithm development or improvement	Offline protocol and dataset protocol	Informed consent and ethics committee	P, FE, C, F
[52]	MI	Algorithm	Algorithm development or improvement	Dataset protocol	No testing applicable due to dataset use	P, FE, C
[53]	MI	Algorithm	Robotic	Online protocol	No information	P, FE, C, F
[54]	MI	Algorithm	Rehabilitation	Dataset protocol	No testing applicable due to dataset use	P, FE, C
[55]	MI	Algorithm	General	Dataset protocol	No testing applicable due to dataset use	P, FE, C
[56]	MI	Methodology/ Concepts	General	No information	No information	P, FE, C, F
[57]	MI	Algorithm	Algorithm development or improvement	Dataset protocol	No testing applicable due to dataset use	P, FE, C, F
[58]	MI	Application	General	Online protocol	Human factor assessment, informed consent, question about BCI experience, Ethic committee	P, FE, C, F
[59]	MI	Algorithm	Rehabilitation	Dataset protocol	No testing applicable due to dataset use	P, FE, C
[60]	SSVEP	Algorithm	Robotic	Online protocol	No information	P, FE, C, F
[61]	MI	Algorithm	Rehabilitation	Dataset protocol	No testing applicable due to dataset use	P, FE, C
[62]	MI	Algorithm	Algorithm development or improvement	Dataset protocol	No testing applicable due to dataset use	P, FE, C
[63]	MI	Application	Robotic	Online and offline protocol	Informed consent, ethics committee	P, FE, C, F
[64]	MI	Application	Robotic	Online and offline protocol	Human factors	P, FE, C, F
[65]	P300, MI	Application	Rehabilitation	Online and offline protocol	Ethics committee, informed consent, clinical examination, human factor assessment	P, FE, C, F
[66]	P300, MI, SSVEP	Methodology/ Concepts	General	Online and offline protocol	No information	No information
[67]	MI	Methodology/ Concepts	Rehabilitation	Online and offline protocol	Ethics committee, informed consent, human factor assessment	P, FE, C, F
[68]	MI	Algorithm	Assistance	Online and offline protocol	Ethics committee, informed consent	P, FE, C, F
[69]	MI	Algorithm	Algorithm development or improvement	Dataset protocol	No testing applicable due to dataset use	P, FE, C
[70]	MI	Algorithm	General	Dataset protocol	No testing applicable due to dataset use	P, FE, C
[71]	MI	Algorithm	Algorithm development or improvement	Dataset protocol and offline protocol	Ethics committee, informed consent, clinical examination, and human factor assessment	P, FE, C
[72]	MI	Algorithm	Rehabilitation	Dataset protocol and offline protocol	Informed consent, ethics committee	P, FE, C

**Table 2:** Color reference for functional and non-functional components.

Category	Color	Description
Planning	Magenta	Intellectual creativity and design
Human	Green	User, what they perceive or do
Physical	Gray	Tangible elements and components
Processing	Blue	Logical data flow and calculations
Learning	Orange	Interpretation and decision-making
Control	Brown	Synchronization and communication with the device or application

**Table 3:** Taxonomy and checklist of the integrated dual framework for BCI.

Domain of Study	Functional Category	Component	Definition	Taxonomy Parameters	Checklist Verification
Experiment	Experimental procedure and ethical considerations	Recruitment and Preliminary Activities	Description of how users are recruited and the activities prior to consolidating the experiment, such as preparing documents describing the experiment and providing informed consent.	Recruitment Experimental document formulation Consent document formulation Permits Document printing	Are the recruitment methods, formulated documents, and ethical permits explicitly described?
Experiment	Experimental procedure and ethical considerations	Preparation and Information	Description of the activities to be performed on the day of the experiment, such as equipment and user setup, signal verification, and signing of the informed consent form.	Experiment information User preparation Emergency protocol Informed consent procedure	Are the user preparation process and the provision of instructions and informed consent documented?
Experiment	User Characterization	Initial tests	Description of initial tests to characterize the user, perform physical tests to conduct the experiment or evaluate the experience with BCI.	Physical fitness Mental fitness BCI experience	Are demographic data, physical/mental fitness and BCI experience reported?
Experiment	Experimental lifecycle	Training session	Stage where user data is recorded, offline analysis is performed, and the BCI system model is adjusted.	Data recording Processing Training Analysis	Are the steps for training session detailed?
Experiment	Experimental lifecycle	Validation session	Stage where the trained model is used and the online experiment is conducted.	Data collection Processing Validation	Are the steps for the validation session detailed?
Experiment	User Characterization	Additional tests	Description of psychological or complementary tests.	Psychological or complementary tests to measure the user’s attention, concentration, or alertness.	Are supplementary cognitive tests evaluated and reported?
Experiment	Experimental lifecycle	Experiment completion	Description of how the experiment will be completed and the final survey.	Removing elements from the user Perception survey Data storage	Is the completion protocol described?
Experiment	Experimental lifecycle	Experiment timeline	Description of the total time required for the experiment.	Time details for each activity during the experiment	Is the exact timeline for each phase detailed?
BCI System	System configuration	Paradigm selection	Description of the paradigm to be used.	Recording method Brain signal Brain region	Are the chosen paradigm and target brain region defined?
BCI System	System configuration	Stimulus type	Description of the system’s tasks.	Type Stimulus generation Display device Task	Are the specific stimulus characteristics defined?
BCI System	System configuration	Environment	Description of the location where the experiment will be conducted.	User conditions Stimulus conditions Site conditions	Are the physical conditions of the experimental site described?
BCI System	System configuration	User	User profile.	User information Initial testing, e.g., user demographics and human factors	Is the user configuration described?
BCI System	Signal acquisition	Interface	Technical description of the interface.	Electrodes Location in brain region Sampling rate	Is the interface used for signal recording specified?
BCI System	Signal acquisition	Data acquisition	Description of the hardware and software.	Hardware Software	Are the hardware and software used for data acquisition specified?
BCI System	Signal processing	Preprocessing	Description of data cleansing and normalization techniques.	Artifacts Filters	Are the data cleaning techniques, artifact rejection methods, and filter parameters detailed?
BCI System	Signal processing	Feature extraction	Description of the extraction techniques used to identify the most relevant features that identify the user’s intent.	Time-domain technique Frequency-domain technique Spatial technique	Are the extraction techniques used to identify the relevant features reported?
BCI System	Signal processing	Classification	Description of the classification techniques and metrics used to evaluate the model and identify the user’s intent.	Machine learning algorithms Evaluation metrics	Are the specific machines learning algorithms and the metrics used to evaluate model performance specified?
BCI System	Application control	Command translation	Description of how the inputs relate to the outputs that will control the device.	Converts classified intent into a specific command to control a device	Is the translation of classified intent into physical commands described?
BCI System	Application control	Device	Description of the application the user will control.	Application	Is the target application described?
BCI System	Application control	Feedback	Description of how the user can determine the success of their intent and make appropriate adjustments.	Visual Auditory Tactile	Is the type of feedback provided to the user to confirm their intention explained?

**Table 4:** Documentation of taxonomy and checklist of the integrated dual framework for BCI.

Taxonomy and Checklist of the Integrated Dual Framework for BCI			[102]	[103]	[104]
Domain of Study	Functional Category	Specific Component			
Experiment	Experimental procedure and ethical considerations	Document approval	Ethical standard of the Universitat de València research committee.	International recommendations.	Ethics committee of the involved institutions.
Experiment	Experimental procedure and ethical considerations	Informed consent	Informed consent from all participants, describe the participant information procedure and EEG headset preparation. Describe experimental procedures such as environment.	Informed consent from all participants indicates that participants received posture instructions. Describe experimental procedures such as environment.	Informed consent from all participants. Describe the preparation of the environment and application of the conductive gel. Describe experimental procedures such as environment.
Experiment	User Characterization	Physical and Cognitive Tests	Questionnaires on daily activities and psychological variables were administered, along with a Jacobson progressive facial relaxation procedure and final test.	Participants were assessed to ensure they had normal or corrected vision and no history of epilepsy or psychotropic medication use.	A history and evaluation of the participants were conducted. Goniometry data from the shoulder, elbow, and forearm were evaluated and a final test performed.
BCI system	Signal Acquisition	Interface	Neuroelectrics’ Enobio amplifier; describe the procedure for the experiment such as timeline and stimulus.	Neuroelectrics’ Enobio amplifier; describe the procedure for the experiment such as timeline and stimulus.	Gtec Nautilus; describe the procedure for the experiment such as timeline and stimulus.
BCI system	Signal Acquisition	Electrode Placement	Channels F3, F4, C3, Cz, C4, T7, T8, and Pz. MI paradigm.	Channels O1, O2, Oz, PO3, PO4, Pz, Cz, and Fz. SSVEP paradigm.	Channels C3, C1, Cz, C2, C4, CP3, CPz, and CP4. MI paradigm.
BCI system	Signal Processing	Filters	A band-pass filter between 2 and 100 Hz with a 50 Hz notch filter was used. A large Laplacian filter was applied for spatial filtering.	A fourth-order Butterworth band-pass filter between 6 and 40 Hz and a 50 Hz notch filter were applied to the EEG signal.	OpenVibe software was used for data collection, processing, and analysis of EEG signals.
BCI system	Signal Processing	Feature extraction	AR model to estimate sensorimotor rhythm amplitudes and calculate power in adjacent 3 Hz bins.	A spatial approach was used for feature extraction. A Common Spatial Pattern (CSP) filter and Band Power were used.	The Filter Bank Common Spatial Pattern (FBCSP) algorithm was used for feature extraction from the acquired signals.
BCI system	Signal Processing	Classification	Linear Discriminant Analysis; describe the comparison of motor imagery strategies to evaluate system performance.	Linear Discriminant Analysis and the results of the online testing phase, including success rates, are analyzed and presented.	Linear Discriminant Analysis and an online testing phase is described.
BCI system	Application	Device	A low-cost robotic guide for arm movements was designed and implemented using 3D-printing techniques for rehabilitation.	A six-axis industrial manipulator, model, TX60 from Stäubli, was used to control a robotic arm.	Rehabilitation tool with a UR3 collaborative robot for upper extremity therapy
BCI system	Application	Feedback	The user obtains movement feedback that reflects their intention, providing visual and somatosensory feedback.	The architecture of the SSVEP BCI control system includes a feedback loop.	The system incorporates visual and auditory feedback to improve motor control. The robot’s movement serves as feedback for the user.

**Table 5:** Detailed independent rater evaluations.

Taxonomy Parameters	Checklist Verification	Article 1 (Rater 1)	Article 2 (Rater 1)	Article 3 (Rater 1)	Article 1 (Rater 2)	Article 2 (Rater 2)	Article 3 (Rater 2)
• Recruitment	Are the recruitment methods, formulated documents, and ethical permits explicitly described?	No	Yes	No	No	Yes	No
• Experimental document formulation							
• Consent document formulation							
• Permits							
• Document printing							
• Experiment information	Are the user preparation process and the provision of instructions and informed consent documented?	No	Yes	No	No	Yes	No
• User preparation							
Emergency protocol							
Informed consent procedure							
• Physical fitness	Are demographic data, physical/mental fitness and BCI experience reported?	No	Yes	No	No	No	No
• Mental fitness							
• BCI experience							
• Data recording	Are the steps for training session detailed?	Yes	Yes	Yes	Yes	Yes	Yes
• Processing							
• Training							
• Analysis							
• Data collection	Are the steps for validation session detailed?	Yes	Yes	Yes	Yes	Yes	Yes
• Processing							
• Validation							
• Psychological or complementary tests to measure the user’s attention, concentration, or alertness.	Are supplementary cognitive tests evaluated and reported?	No	Yes	No	No	No	No
• Removing elements from the user	Is the completion protocol described?	No	No	No	No	No	No
• Perception survey							
• Data storage							
• Time details for each activity during the experiment	Is the exact timeline for each phase detailed?	Yes	Yes	Yes	No	Yes	No
• Recording method	Are the chosen paradigm and target brain region defined?	Yes	Yes	Yes	Yes	Yes	Yes
• Brain signal							
• Brain region							
• Type	Are the specific stimulus characteristics defined?	Yes	Yes	Yes	No	Yes	No
• Stimulus generation							
• Display device							
• Task							
• User conditions	Are the physical conditions of the experimental site described?	No	Yes	No	No	No	No
• Stimulus conditions							
• Site conditions							
• User information	Is the user configuration described?	No	Yes	No	No	Yes	No
• Initial testing, e.g., user demographics and human factors							
• Electrodes	Is the interface used for signal recording specified?	Yes	Yes	Yes	Yes	Yes	Yes
• Location in brain region							
• Sampling rate							
• Hardware	Are the hardware and software used for data acquisition specified?	No	Yes	No	No	Yes	No
• Software							
• Artifacts	Are the data cleaning techniques, artifact rejection methods, and filter parameters detailed?	Yes	Yes	Yes	Yes	Yes	Yes
• Filters							
• Time-domain technique	Are the extraction techniques used to identify the relevant features reported?	Yes	Yes	Yes	Yes	Yes	Yes
• Frequency-domain technique							
• Spatial technique							
• Machine learning algorithms	Are the specific machine learning algorithms and the metrics used to evaluate model performance specified?	Yes	Yes	Yes	Yes	Yes	Yes
• Evaluation metrics							
• Converts classified intent into a specific command to control a device	Is the translation of classified intent into physical commands described?	No	Yes	No	No	Yes	No
• Application	Is the target application described?	Yes	Yes	Yes	No	Yes	No
• Visual	Is the type of feedback provided to the user to confirm their intention explained?	No	Yes	No	No	Yes	No
• Auditory							
• Tactile							

**Table 6:** Item-level mapping of the proposed integrated dual framework against existing BCI models and standards.

Feature	Mason & Birch	IEEE P2731/P2794	TOBI/BNCI	Proposed Integrated Dual Framework
Foundational technical pipeline	✓	✓	✓	✓ (Adopted)
Standardization of unified data formats & ontology	x	✓	x	x
Hardware interoperability & strategic roadmaps	x	x	✓	x
Broad in vivo neuroethics guidelines	x	✓	✓	✓ (Aligned)
Formalization of non-technical phases	x	✓	✓	✓ (Integrated)
Reporting Checklist	x	x	x	✓ (Contribution)
Visual communication strategy	x	x	x	✓ (Contribution)
Domain-specific constraint (MI/SSVEP in rehabilitation and robotics)	x	x	x	✓ (Targeted scope)

**Figure A1:**
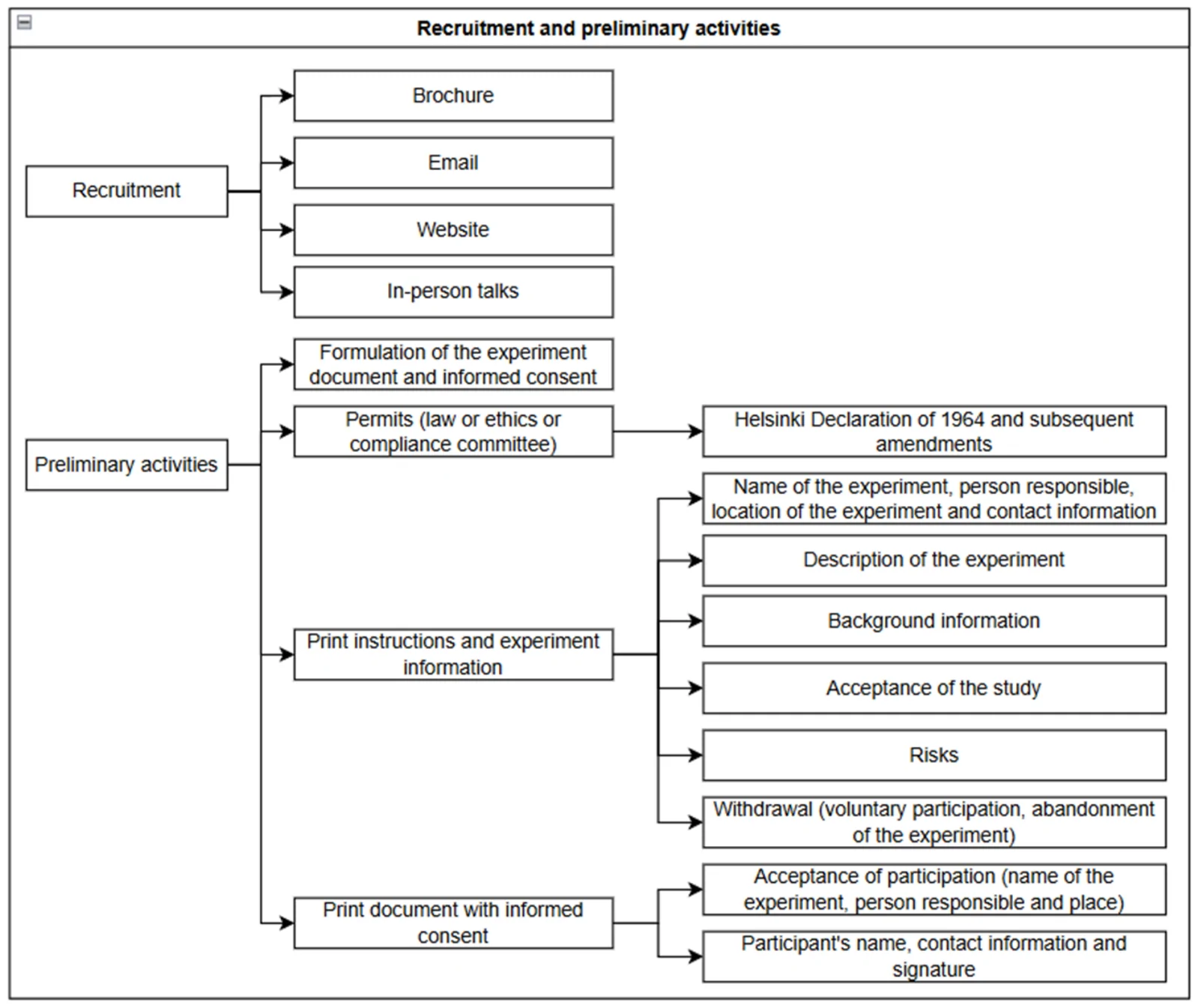
Block diagram of recruitment and preliminary activities.

**Figure A2:**
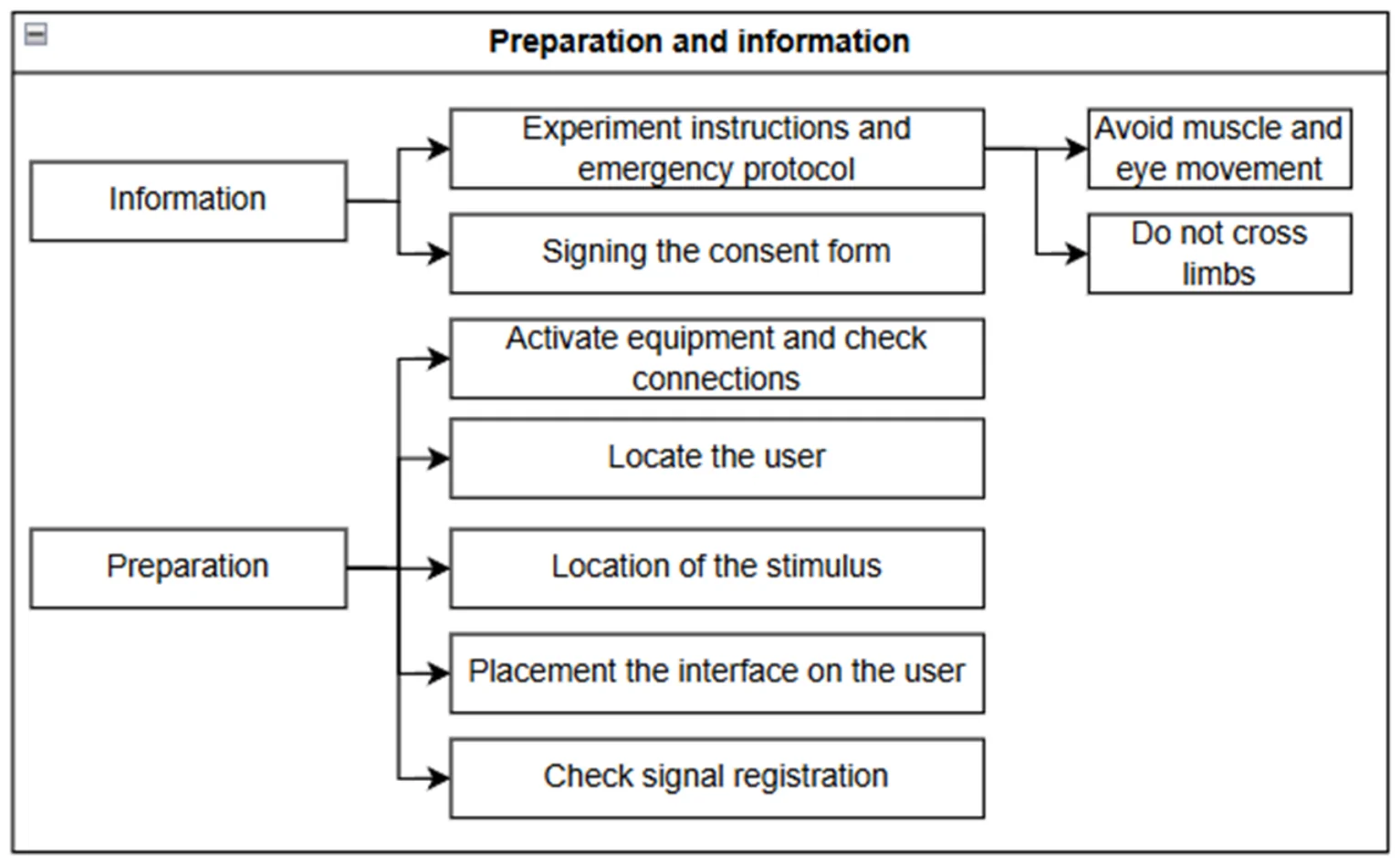
Block diagram of preparation and information.

**Figure A3:**
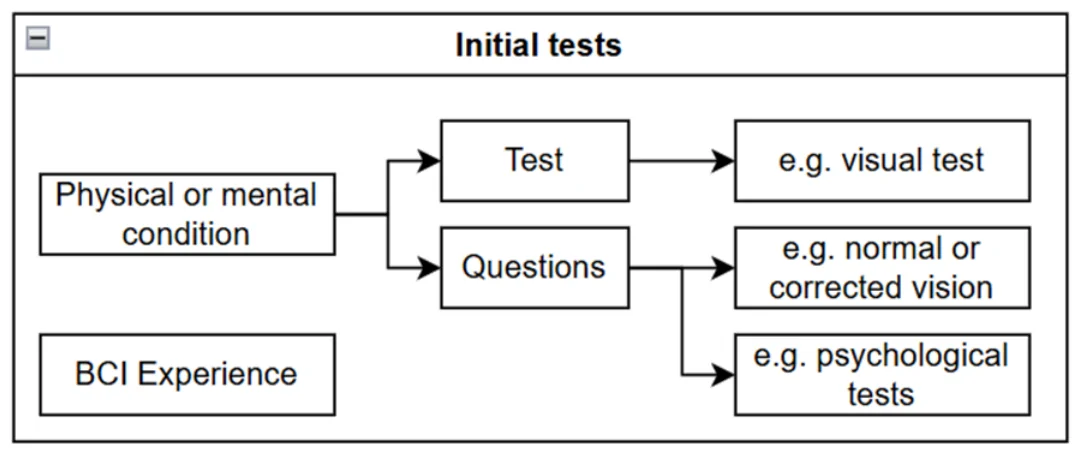
Block diagram of initial test.

**Figure A4:**
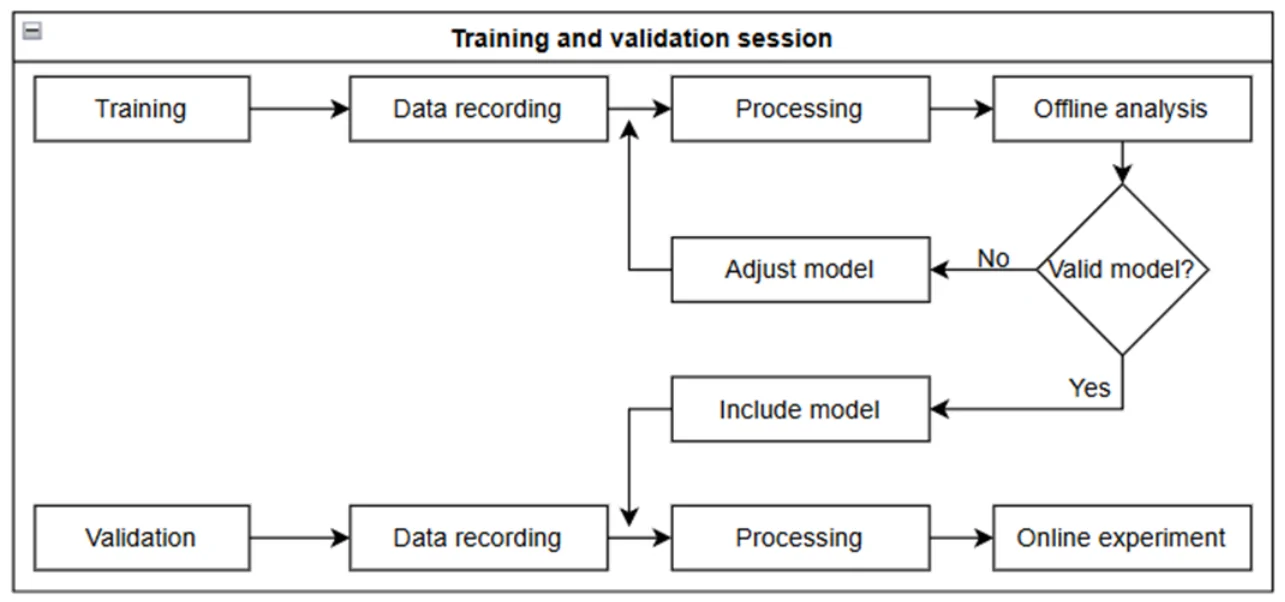
Block diagram of the training and validation session.

**Figure A5:**
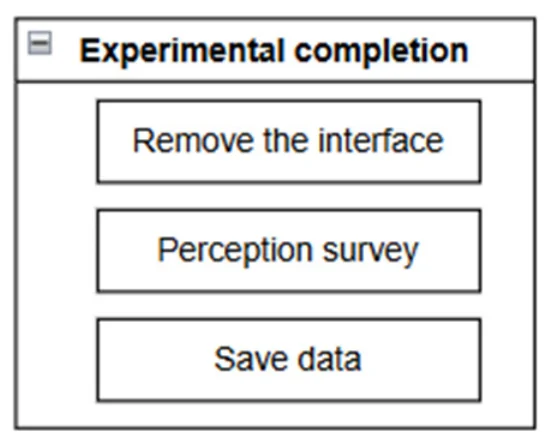
Block diagram of experiment completion.

**Figure A6:**
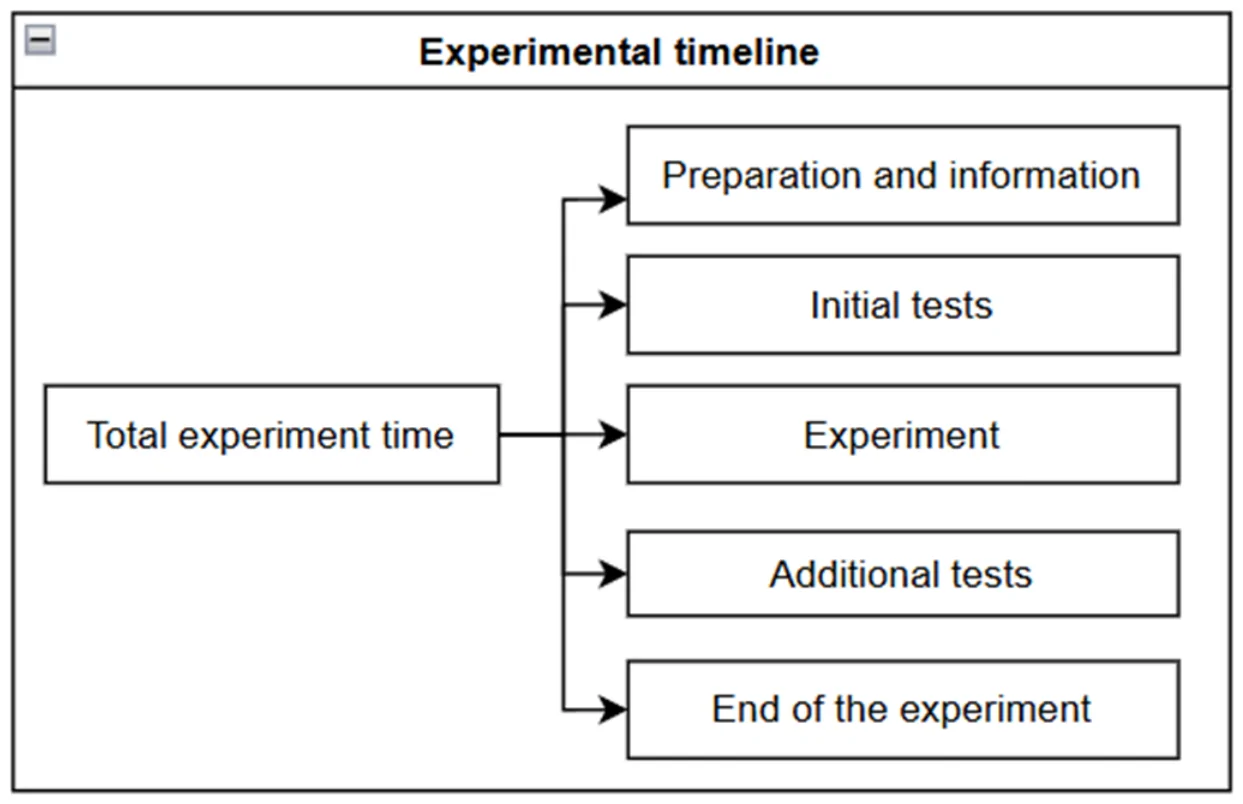
Block diagram of the experiment timeline.

**Figure A7:**
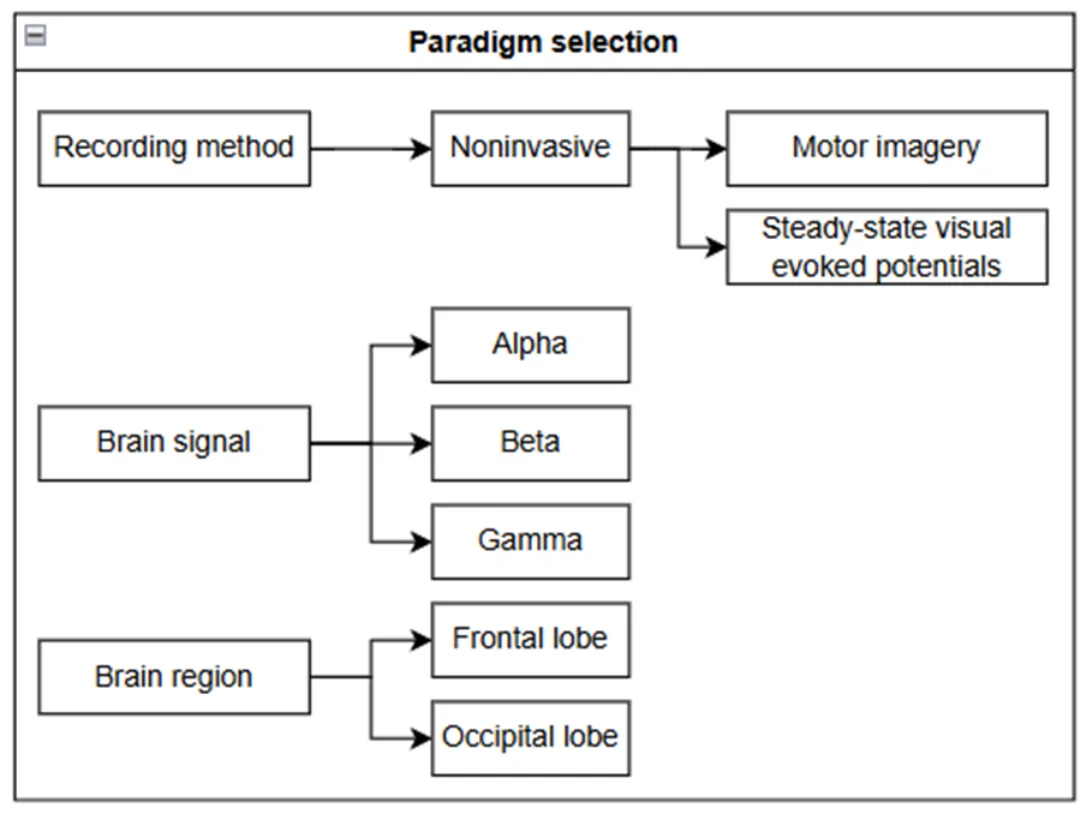
Block diagram of paradigm selection.

**Figure A8:**
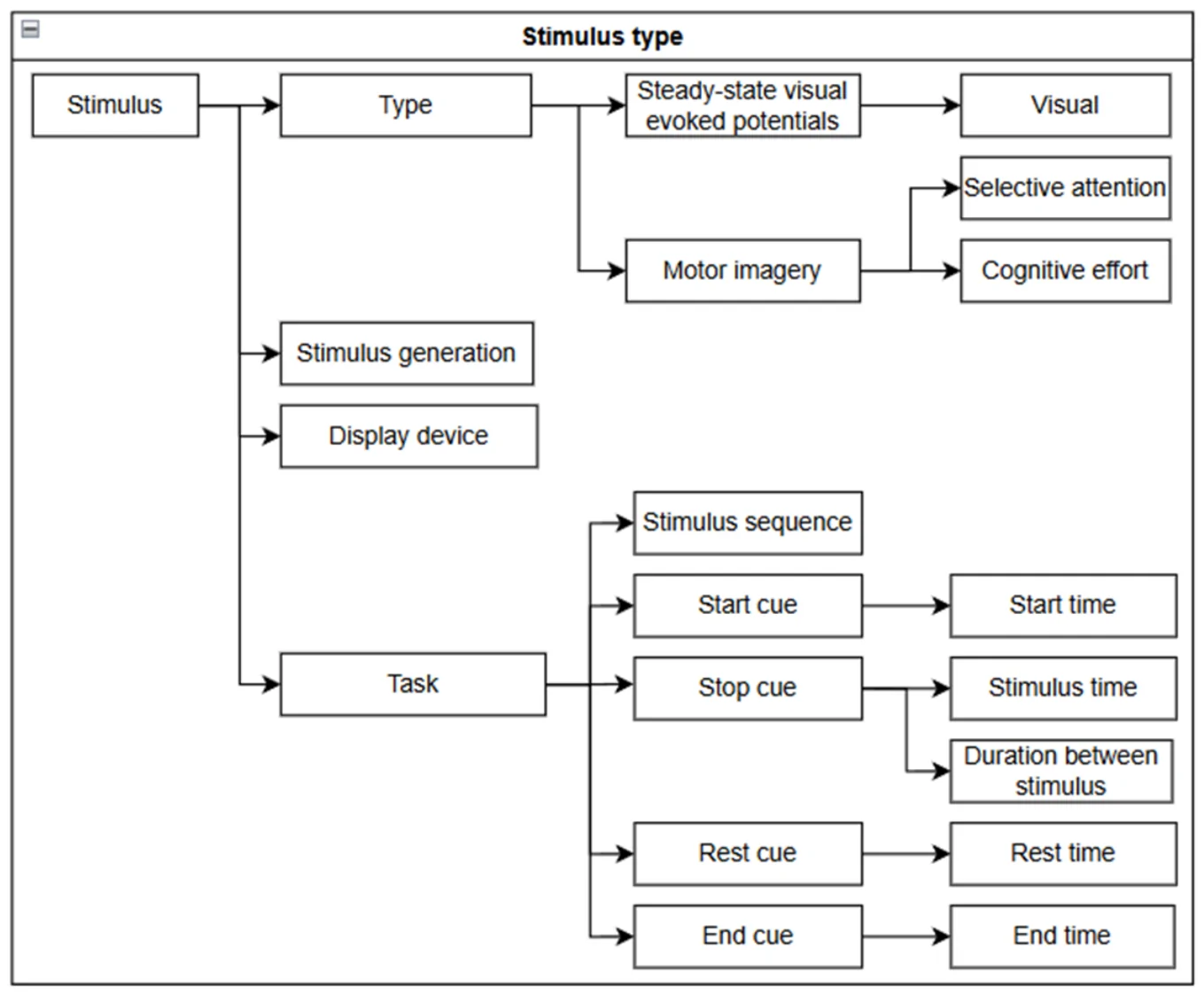
Block diagram of the stimulus type.

**Figure A9:**
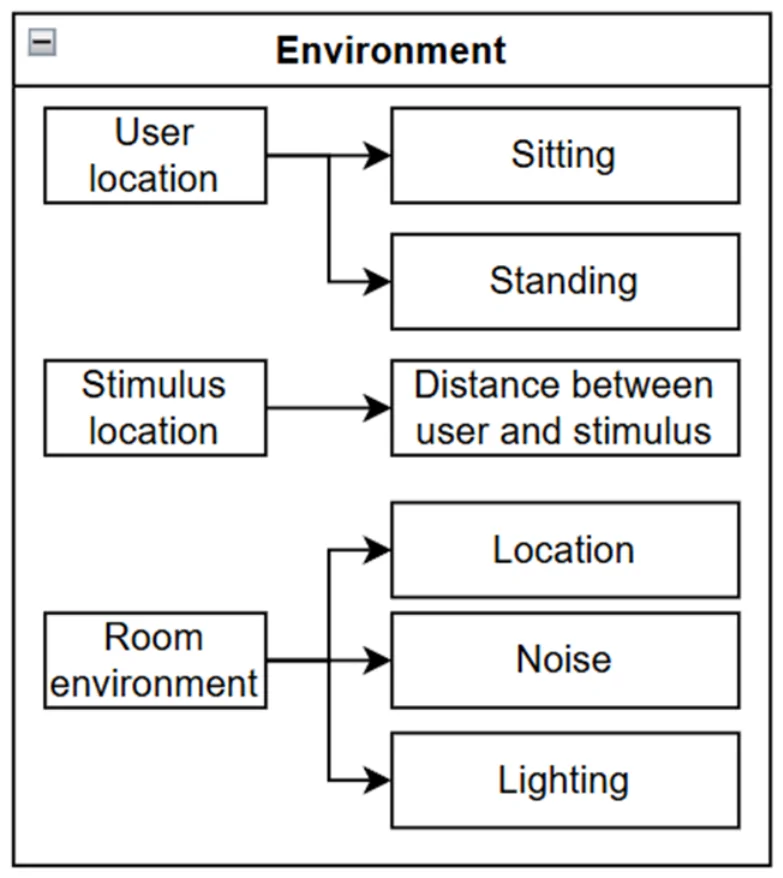
Block diagram of the environment.

**Figure A10:**
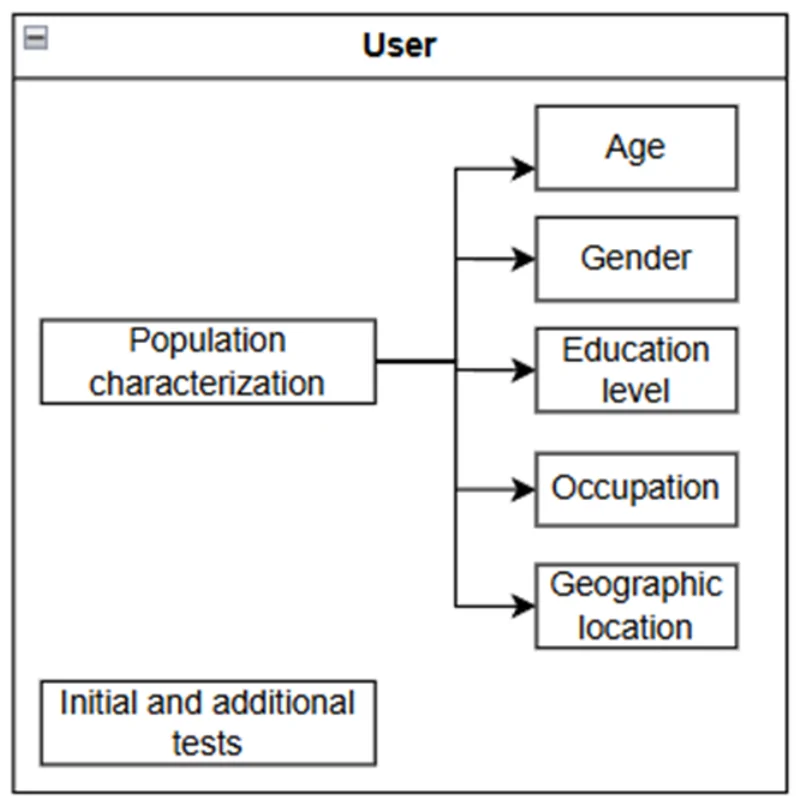
Block diagram of the user.

**Figure A11:**
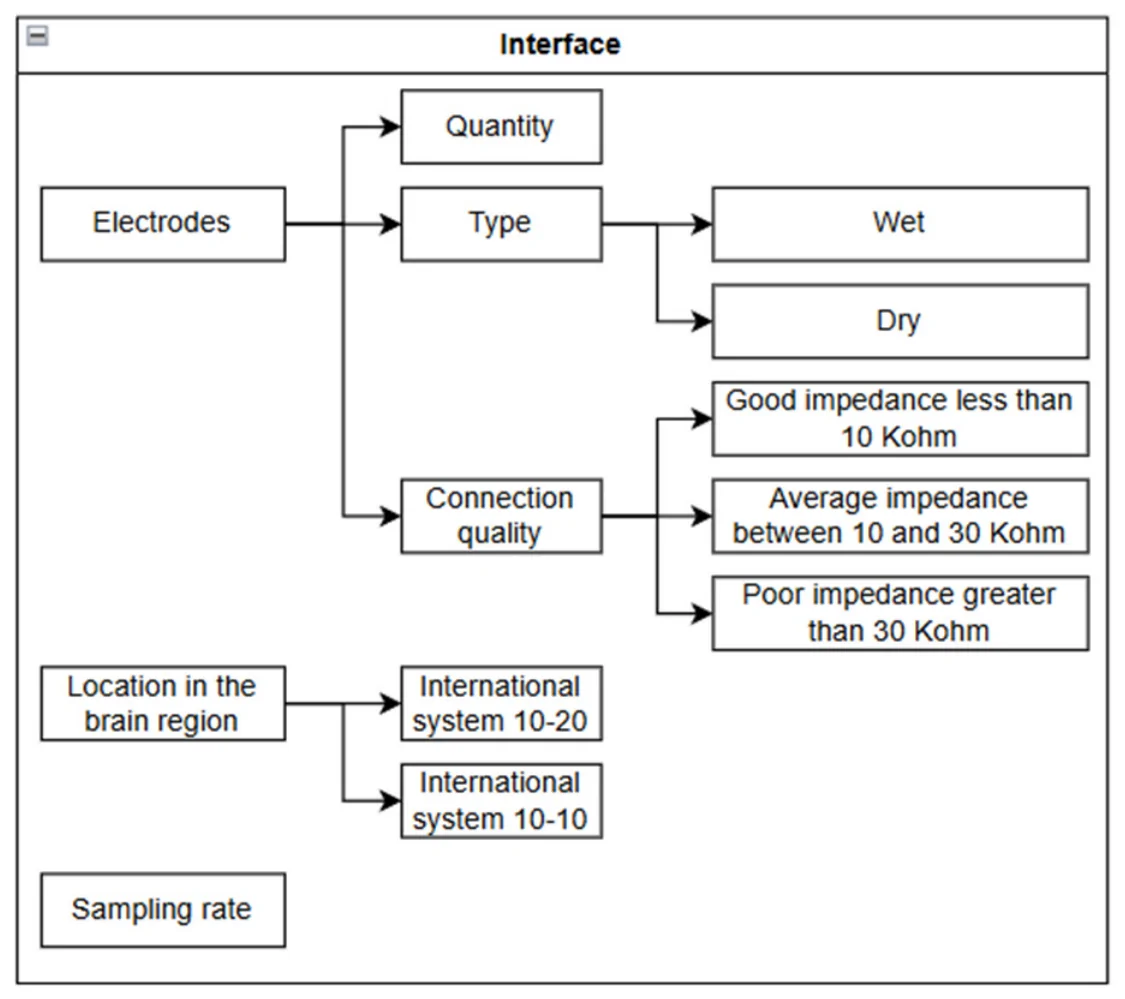
Block diagram of the interface.

**Figure A12:**
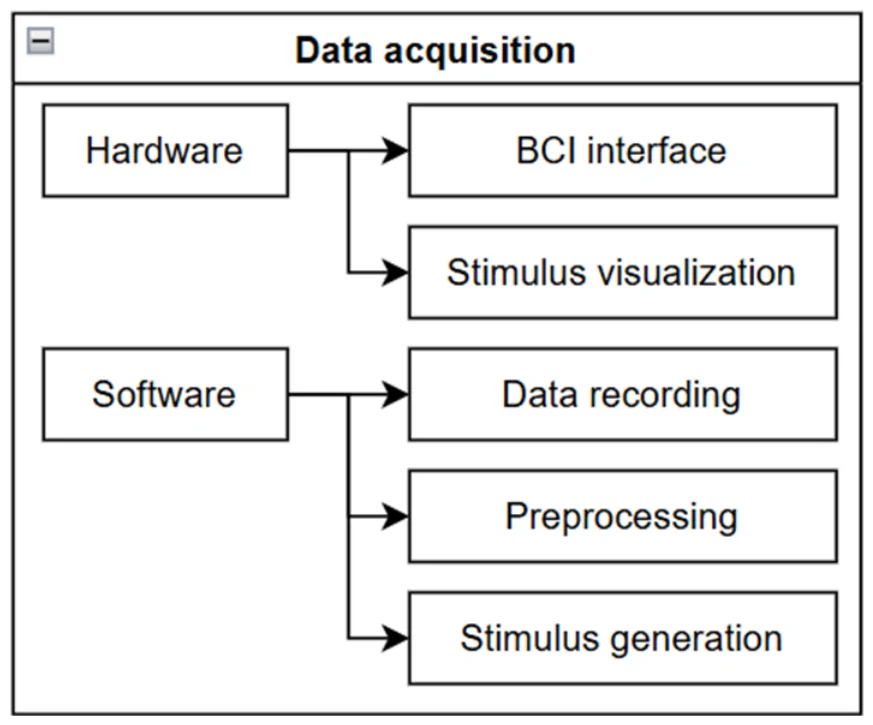
Block diagram of data acquisition.

**Figure A13:**
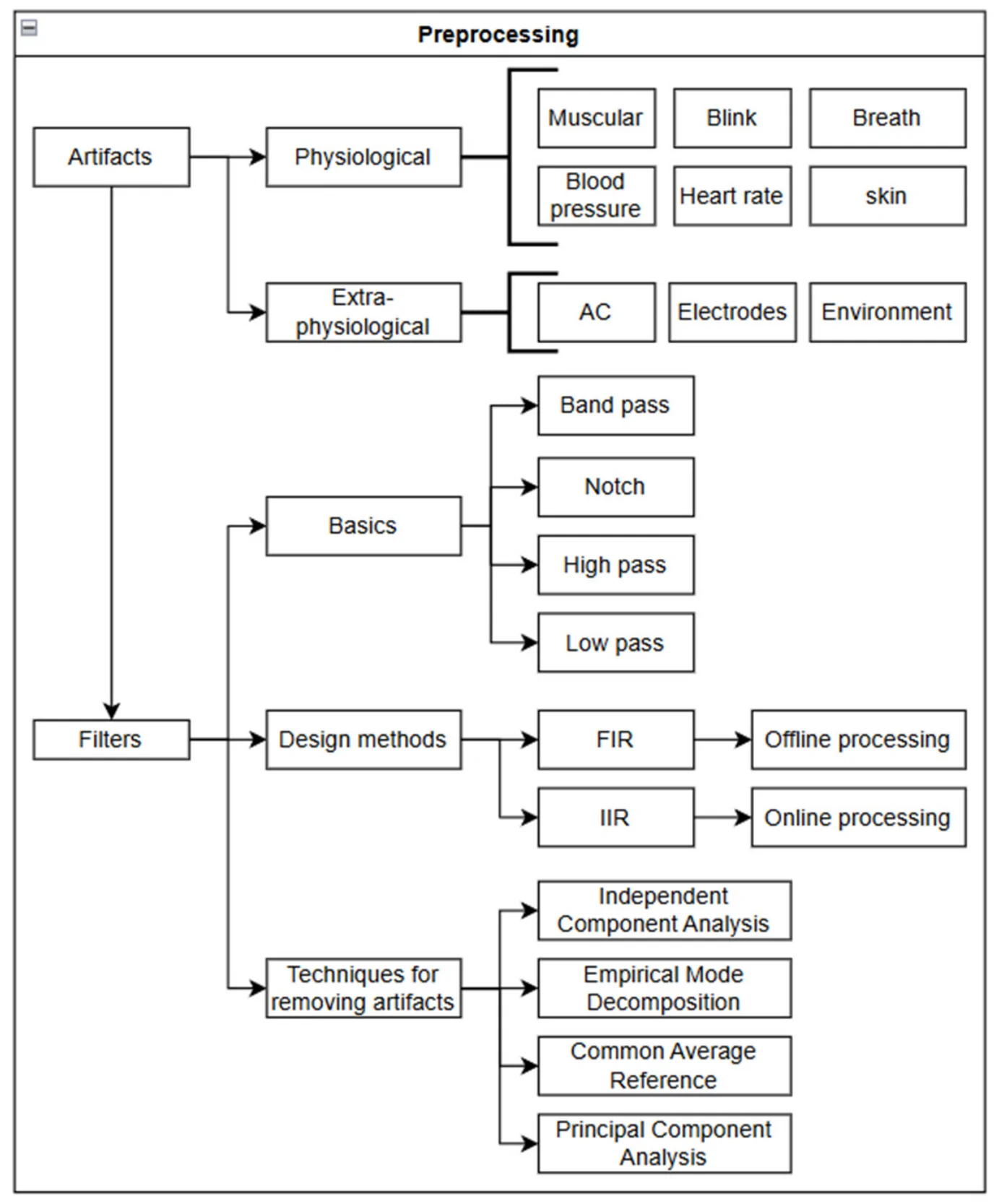
Block diagram of preprocessing.

**Figure A14:**
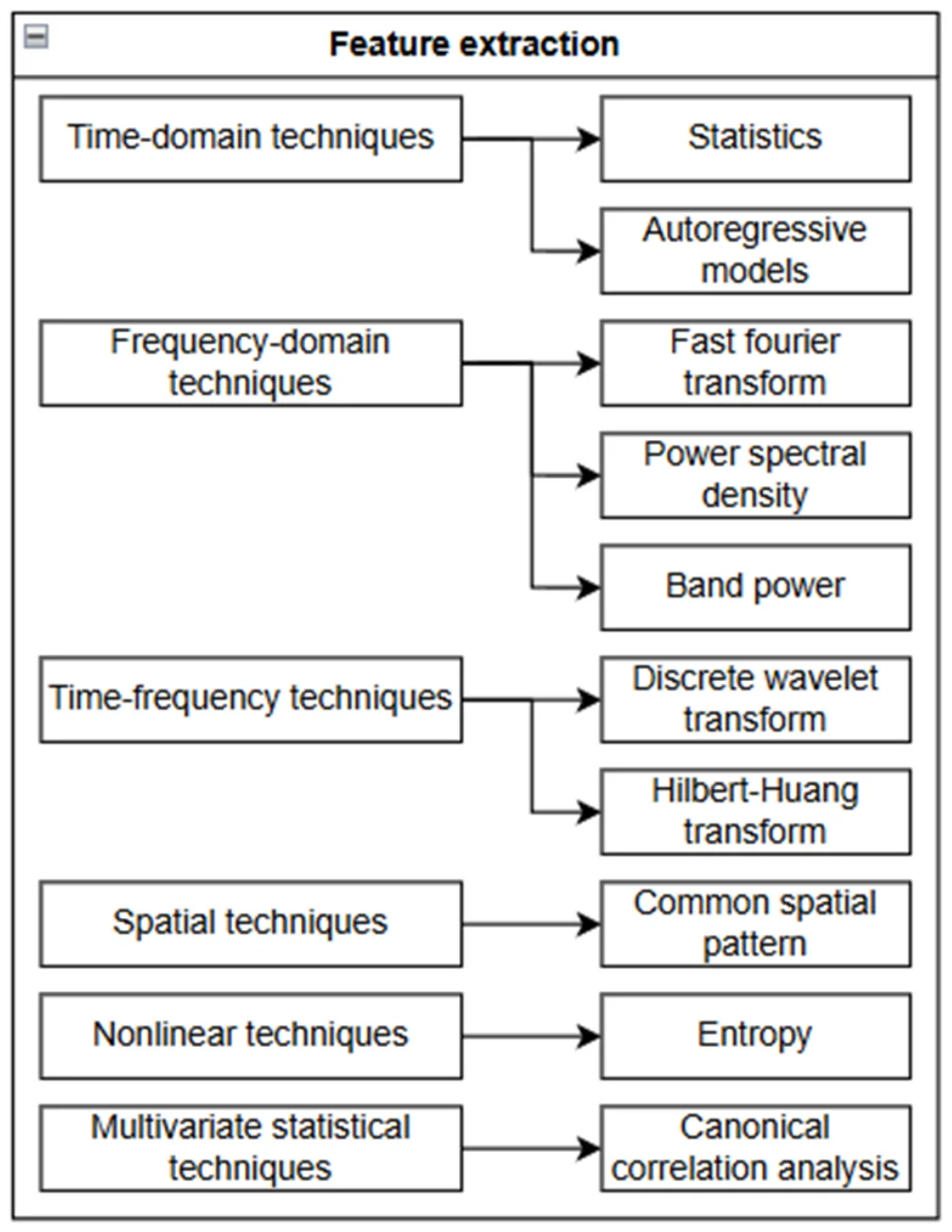
Block diagram of feature extraction.

**Figure A15:**
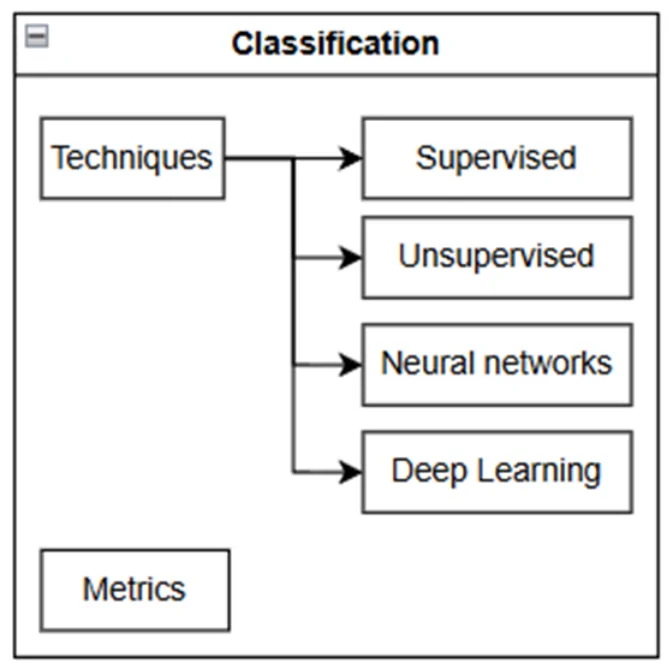
Block diagram of classification.

**Figure A16:**
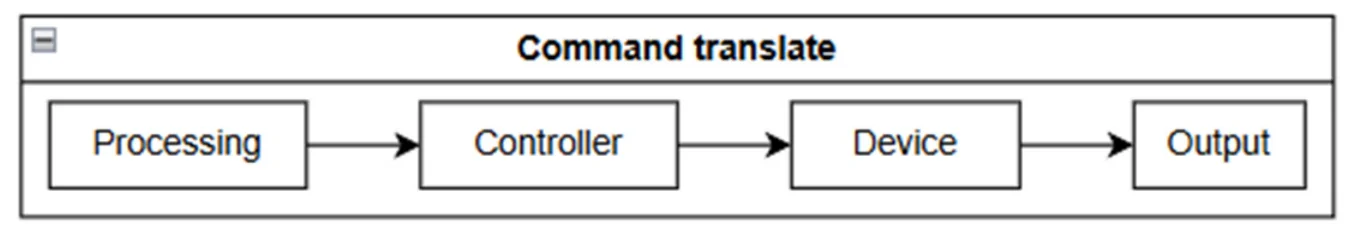
Block diagram of command translation.

## Data Availability

No new data were created or analyzed in this study. Data sharing is not applicable to this article.

## Supplementary Materials

The following supporting information can be downloaded at: https://www.mdpi.com/article/10.3390/s26216764/s1, Table S1: PRISMA Checklist.

## Abbreviations

The following abbreviations are used in this manuscript:

BCI	Brain–computer interfaces
MI	Motor imagery
SSVEP	Steady-State Visually Evoked Potential
EEG	Electroencephalography
LCD	Liquid Crystal Display
LED	Light emitting diodes
RGB	Red, Green, Blue
CSP	Common Spatial Patterns

## Appendix A

Figures for the components of the experimental section are presented: recruitment, preparation and information, initial tests, training session and validation session, experiment completion, and timeline.

## Appendix B

Figures for the components of the BCI system section are presented: paradigm selection, stimulus, environment, user, interface, data acquisition, preprocessing, feature extraction, classification, and command.
